# Detecting early myocardial ischemia in rat heart by MALDI imaging mass spectrometry

**DOI:** 10.1038/s41598-021-84523-z

**Published:** 2021-03-04

**Authors:** Aleksandra Aljakna Khan, Nasim Bararpour, Marie Gorka, Timothée Joye, Sandrine Morel, Christophe A. Montessuit, Silke Grabherr, Tony Fracasso, Marc Augsburger, Brenda R. Kwak, Aurélien Thomas, Sara Sabatasso

**Affiliations:** 1 University Centre of Legal Medicine, Lausanne-Geneva, Rue Michel-Servet 1, 1211 Geneva, Switzerland; 2 University Centre of Legal Medicine, Lausanne-Geneva, Rue Vulliette 04, 1000 Lausanne, Switzerland; 3 Faculty of Biology and Medicine, Lausanne University Hospital, University of Lausanne, Rue Vulliette 04, 1000 Lausanne, Switzerland; 4 Ecole Des Sciences Criminelles/School of Criminal Justice, Faculty of Law, Criminal Justice, and Public Administration, University of Lausanne, 1015 Lausanne-Dorigny, Switzerland; 5 Department of Pathology and Immunology, University of Geneva, Rue Michel-Servet 1, 1211 Geneva, Switzerland

**Keywords:** Diagnostic markers, Myocardial infarction, Metabolomics, Cardiovascular models, High-throughput screening, Mass spectrometry

## Abstract

Diagnostics of myocardial infarction in human post-mortem hearts can be achieved only if ischemia persisted for at least 6–12 h when certain morphological changes appear in myocardium. The initial 4 h of ischemia is difficult to diagnose due to lack of a standardized method. Developing a panel of molecular tissue markers is a promising approach and can be accelerated by characterization of molecular changes. This study is the first untargeted metabolomic profiling of ischemic myocardium during the initial 4 h directly from tissue section. Ischemic hearts from an ex-vivo Langendorff model were analysed using matrix assisted laser desorption/ionization imaging mass spectrometry (MALDI IMS) at 15 min, 30 min, 1 h, 2 h, and 4 h. Region-specific molecular changes were identified even in absence of evident histological lesions and were segregated by unsupervised cluster analysis. Significantly differentially expressed features were detected by multivariate analysis starting at 15 min while their number increased with prolonged ischemia. The biggest significant increase at 15 min was observed for m/z 682.1294 (likely corresponding to S-NADHX—a damage product of nicotinamide adenine dinucleotide (NADH)). Based on the previously reported role of NAD^+^/NADH ratio in regulating localization of the sodium channel (Na_v_1.5) at the plasma membrane, Na_v_1.5 was evaluated by immunofluorescence. As expected, a fainter signal was observed at the plasma membrane in the predicted ischemic region starting 30 min of ischemia and the change became the most pronounced by 4 h. Metabolomic changes occur early during ischemia, can assist in identifying markers for post-mortem diagnostics and improve understanding of molecular mechanisms.

## Introduction

Myocardial infarction (MI) is a leading cause of death and disability. It is defined as cardiac damage caused by myocardial ischemia due to an inadequate or interrupted blood flow to the heart^1,2^. Diagnostics of MI has important implications in clinical settings for survival and treatment of patients and in medico-legal situations for assisting in legal cases and counselling surviving families. The diagnostics relies on showing evidence of ischemia-induced myocardial damage. In hospitals, it can be accomplished during the initial few hours of ischemia using a combination of symptoms, observations in electrocardiogram, circulating biomarkers, and advanced imaging techniques. However, post-mortem morphological diagnostics of MI can be achieved only if ischemia persisted for at least 6–12 h by demonstrating presence of certain morphological features in myocardial tissue using standard histological staining, namely polymorphonuclear leukocytes, pyknosis of nuclei, and hypereosinophilic cardiomyocytes^3,4^. The period during the initial 4 h is referred by pathologists as early myocardial ischemia (EMI). A standardized method for EMI diagnostics in human post-mortem samples is still lacking (especially for the initial 2 h): the nearly normal appearance of myocardium by both gross examination and histology makes it challenging to provide accurate evidence of ischemia-induced myocardial damage^3,4^. Identification of specific and sensitive molecular tissue markers could improve post-mortem diagnostics and can be achieved by characterizing molecular changes in ischemic myocardium. In addition, knowledge about molecular changes could advance our understanding of the mechanisms during EMI.

Metabolomics emerged as a molecular method complementary to genomics, transcriptomics, and proteomics^5^. Metabolomics concerns itself with measurement and characterization of small molecules with molecular weight less than 1500 Da but with diverse polarities, molecular mass, and concentration^5^. The technological and bioinformatical advancements allow performing high-throughput evaluation of metabolites as well as mapping them to corresponding pathways^6^. Metabolomic changes reflect the dynamic reaction at molecular level to perturbations and are the earliest-described responses to ischemia. For example, depletion of high energy phosphates and switch to anaerobic glycolysis occur within minutes of ischemia onset^7^. The majority of high-throughput metabolic profiling in the context of EMI and MI has so far been done in biological fluids (e.g. serum, plasma, whole blood) for discovery of clinical biomarkers^6,8,9^. The metabolome of myocardial tissue was investigated by several studies but primarily in healthy myocardium or in myocardium suffering from heart failure, ischemia/reperfusion, or other cardiac pathology^6,10–14^. Only one study evaluated specifically early ischemia-induced cardiac metabolomic changes (1 h, 1 d, and 10 d) using lysed tissues and ultra-performance liquid chromatography quadrupole time-of-flight mass spectrometry^15^. In fact, many of metabolomics methods require lysing of tissue, which leads to loss of molecules’ spatial localization within the sample^16^.

Recently, matrix assisted laser desorption/ionization imaging mass spectrometry (MALDI IMS) emerged as an innovative approach for high-throughput and label-free metabolomic profiling^17,18^. Unlike other untargeted and conventional MS-based methods, MALDI IMS allows sampling directly from tissue section without digesting it and preserves the capability to visualize metabolite’s distribution in the same tissue section by creating an image reconstruction. The latter is possible because the sample is analysed in a coordinated scanning-like manner: both mass spectra and their corresponding spatial positions (X,Y) are collected at each analysed tissue spot. To date, MALDI IMS has been employed to investigate MI in only 4 studies. The first investigation used this method to evaluate expression of ephrin A1 (a tyrosine kinase receptor) in mice exposed to 4-day ischemia^19^. The second study utilized MALDI IMS for proteomics in one human post-mortem sample^20^. Unfortunately, estimating the duration of ischemia in human post-mortem samples is a challenging task. Lastly, 2 groups applied MALDI IMS to characterize metabolomic changes in animal cardiac tissue exposed to 1-day ischemia^21,22^. We focused on profiling temporal metabolic changes by MALDI IMS in rat myocardium during the initial 4 h of ischemia. An animal model of left anterior descending (LAD) coronary artery ligation was intentionally used to control the start and duration of ischemia, which is impossible to do in human post-mortem samples.

## Results

### Detection of ischemic region by untargeted clustering

**Figure 1:**
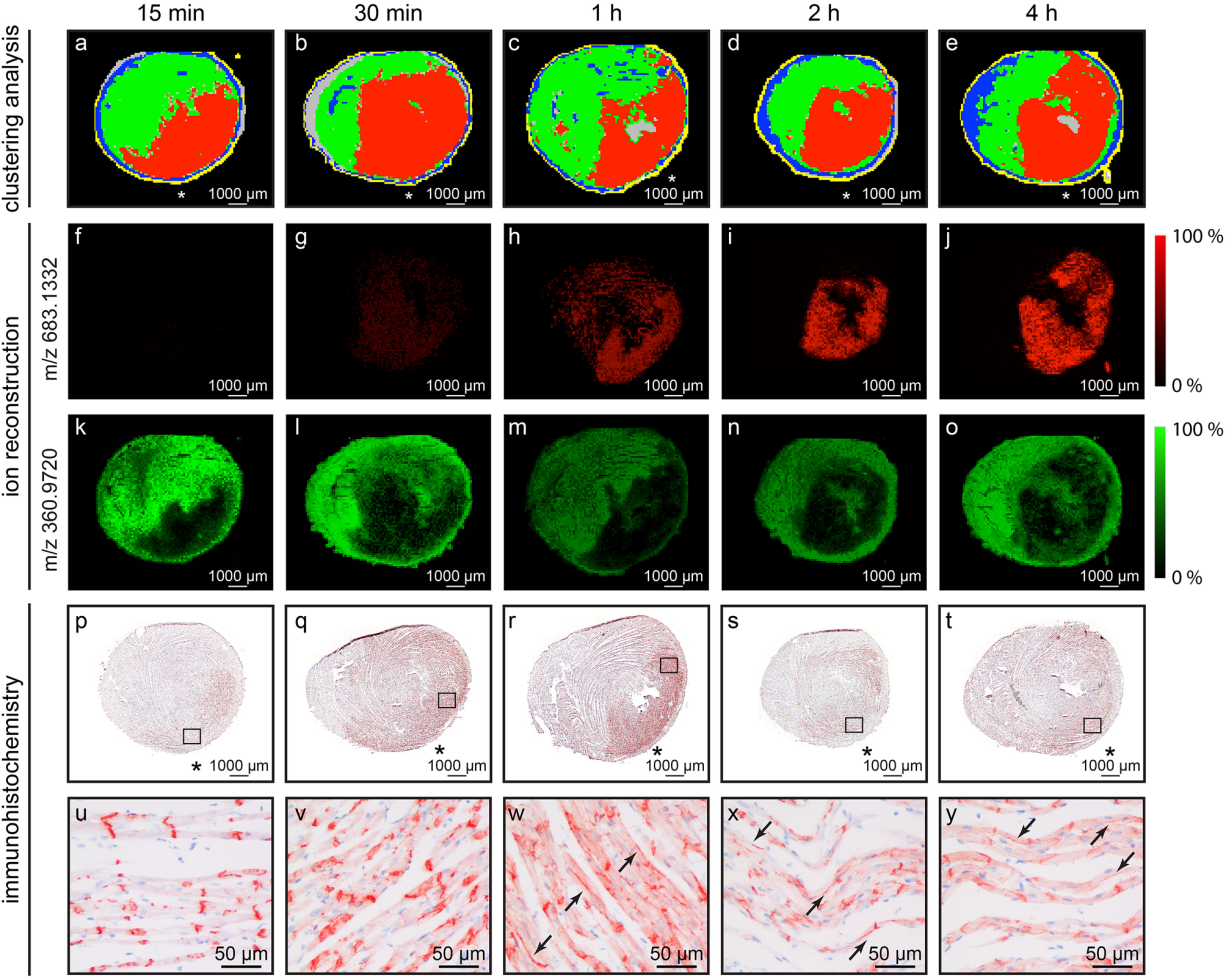
User-independent segmentation of the data separated the predicted ischemic region into its own cluster and this ischemic cluster corresponded to area positive for Cx43 changes. (**a**–**e**) Diagrams of cluster analysis (k = 6) from selected samples at 15 min, 30 min, 1 h, 2 h, and 4 h ischemia; colours represent clusters; n = 5 per time point (biological replicates); red cluster corresponds to the predicted ischemic area; asterisks (*) indicate anterior left ventricle. (**f**–**j**) Reconstructed ionic image from selected samples showing distribution and intensity of m/z 683.1332 (non-annotated feature in the ischemic red cluster); n = 5 per time point (biological replicates); abundance is represented by colour: black and red corresponding to low and high abundance, respectively. (**k**–**o**) Reconstructed ionic image from selected samples showing distribution and intensity of m/z 360.9720 (non-annotated feature in the non-ischemic green cluster); abundance is represented by colour: black and green corresponding to low and high abundance, respectively. (**p**–**y**) Change in Cx43 localization in response to ischemia: (**p**–**t**) Overview of entire cross sections from hearts exposed to 15 min, 30 min, 1 h, 2 h, and 4 h ischemia stained by immunohistochemistry using an antibody that recognizes multiple phospho-forms of Cx43; (**u**–**y**) View at higher magnification of the area highlighted by black rectangles in (**p**–**t**); lateralization and increased cytoplasmic staining was observed starting 1 h; arrows indicate localization of Cx43 to the lateral side of cardiomyocytes.

Data were analysed with a user-independent, unbiased statistical approach, which is called unsupervised cluster analysis^23,24^. Tissue was segmented into regions (clusters) based on multivariate molecular patterns, which were present in the metabolomics data^25,26^. Segmentation of the data into 6 clusters resulted in separation of the predicted ischemic region into a separate cluster as early as 15 min of ischemia and also at all the other ischemic times (Fig. 1a–e). An example of a feature in this ischemic cluster is a molecule with m/z 683.1332: its intensity gradually increased with prolonged ischemia in the predicted ischemic area (Fig. 1f–j). Cluster corresponding to the presumed non-ischemic region contained molecules such as m/z 360.9720, which were expressed in non-ischemic area but decreased in the ischemic area (Fig. 1k–o). Top discriminating features of each cluster are listed in Supplementary Table S3 online.

Previously, EMI was shown to change localization of the gap junctional protein Cx43. While Cx43 is present primarily at the intercalated discs in a healthy heart, prolonged ischemia leads to its redistribution within the cardiomyocytes: first, it relocates from intercalated discs to the lateral side of the cardiomyocyte and, subsequently, it is internalized into the cytoplasm^27^. Immunohistochemistry for Cx43 was used to verify the presence of ischemic region in our samples, i.e. in order to confirm the successful provocation of ischemia in our experimental model. The cluster in the predicted ischemic area generally corresponded well with the region positive for Cx43 redistribution as detected by immunohistochemistry, i.e. the signal was originating primarily from the antero-lateral left ventricular wall (Fig. 1p–y).

### Metabolic changes induced by ischemia

**Figure 2:**
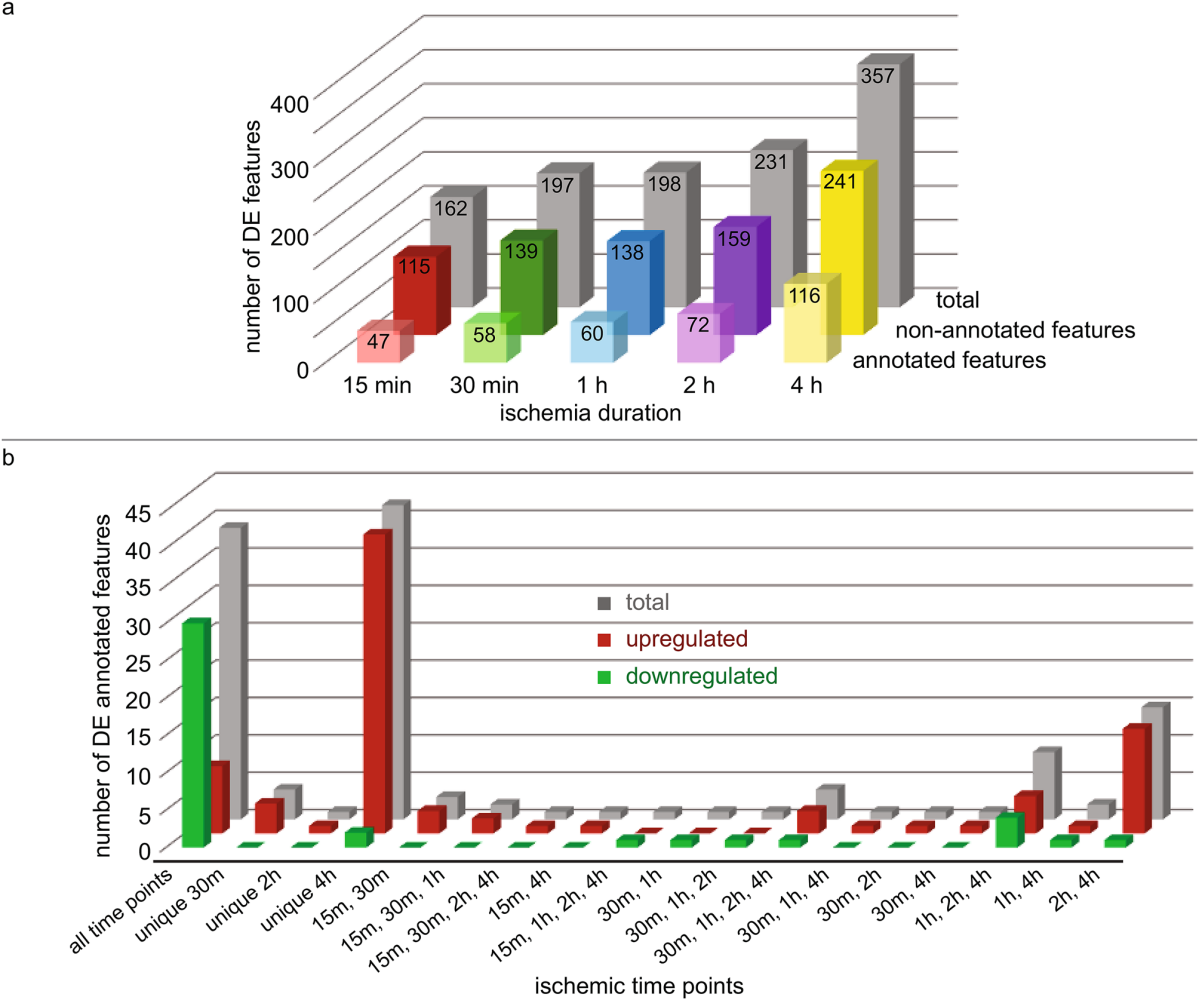
Number of annotated and non-annotated DE features. (**a**) Number of DE features at each time point: the number of both annotated and non-annotated features was increased with prolonged ischemia. (**b**) Number of annotated DE features according to temporal expression pattern and direction of regulation (up or down): changed at all time points, unique to a given time point, or differentially expressed at multiple ischemic timepoints.

Data were extracted from a region located in the predicted ischemic cluster (ROI 1, Supplementary Fig. S1c online) and a multivariate analysis was performed between control and ischemic samples at each time point in order to determine the significant DE features. Consistently with the clustering analysis, the differential expression in the ischemic region was observed as early as 15 min of ischemia (Fig. 2a, Supplementary Table S4 online). Thereafter, the total number of DE features increased with the highest number of DE features at 4 h. Approximately 29–33% of DE features at each ischemic time point were matched to a known metabolite using a monoisotopic mass matching within a window of  ± 10 ppm (referred to as annotated DE features throughout this article). Many of these annotated DE features were previously detected with 9-aminoacridine matrix, which further supports the validity of our method and findings^13,28^.

**Figure 3:**
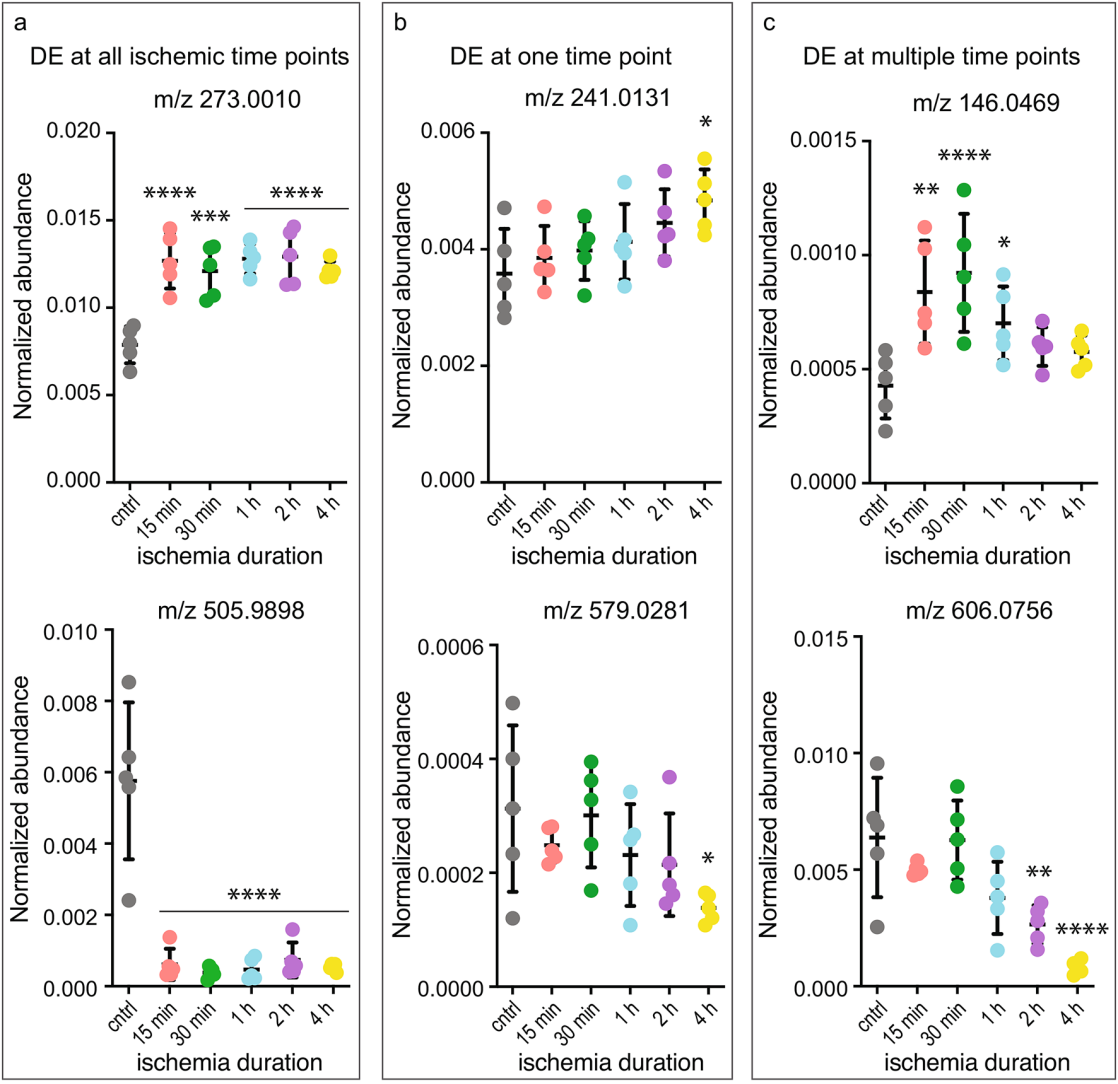
Temporal patterns of selective annotated DE features (mean normalized abundance from ROI 1 ± SD). Features were differentially expressed either (**a**) at all time points: m/z 273.0010 (possibly d-glucuronic acid 1-phosphate) and m/z 505.9898 (possibly ATP 505.9898), up- and down-regulated respectively, (**b**) only at one time point: m/z 241.0131 (possibly inositol cyclic phosphate) and m/z 579.0281 (possibly UDP-glucuronic acid), up- and down-regulated respectively, or (**c**) at multiple time points: m/z 146.0469 (possibly l-glutamic acid) and m/z 606.0756 (possibly UDP-N-acetylglucosamine), up- and down-regulated respectively; n = 5 per time point (biological replicates); statistical significance was determined by multivariate regression analysis using Benjamini–Hochberg’s FDR (*P < 0.05; **P < 0.01; ***P < 0.001; ****P < 0.0005).

Various temporal patterns were evident among DE features: while some features were DE at all of the time points (e.g. m/z 273.0010—possibly corresponding to D-glucuronic acid 1-phosphate and m/z 505.9898—possibly corresponding to ATP), others were either unique to a given time point (e.g. m/z 241.0131—possibly corresponding to inositol cyclic phosphate and m/z 579.0281—possibly corresponding to UDP-glucuronic acid), or DE at multiple time points (but not all 5 time points, e.g. m/z 146.0469—possibly corresponding to L-glutamic acid and m/z 606.0756—possibly corresponding to UDP-N-acetylglucosamine) (Figs. 2b and 3). Annotated DE features common to all time points were mostly decreased, except the 9 increased annotated DE features (Fig. 2b). The highest number of upregulated annotated features were unique to 4 h but a few were also upregulated at other time points. Several of the annotated DE features have been previously implicated in response to ischemia (e.g. ATP), which was an additional confirmation that the MALDI IMS is a suitable method for evaluation of metabolomic changes. At the same time, novel annotated and non-annotated DE features were also identified, which were not previously known to respond to ischemia (Supplementary Table S4 online). None of the features were DE in the non-ischemic region (ROI 2, Supplementary Fig. S1c online) at FDR < 0.05 (data not shown).

### Ischemic changes during the first hour

**Figure 4:**
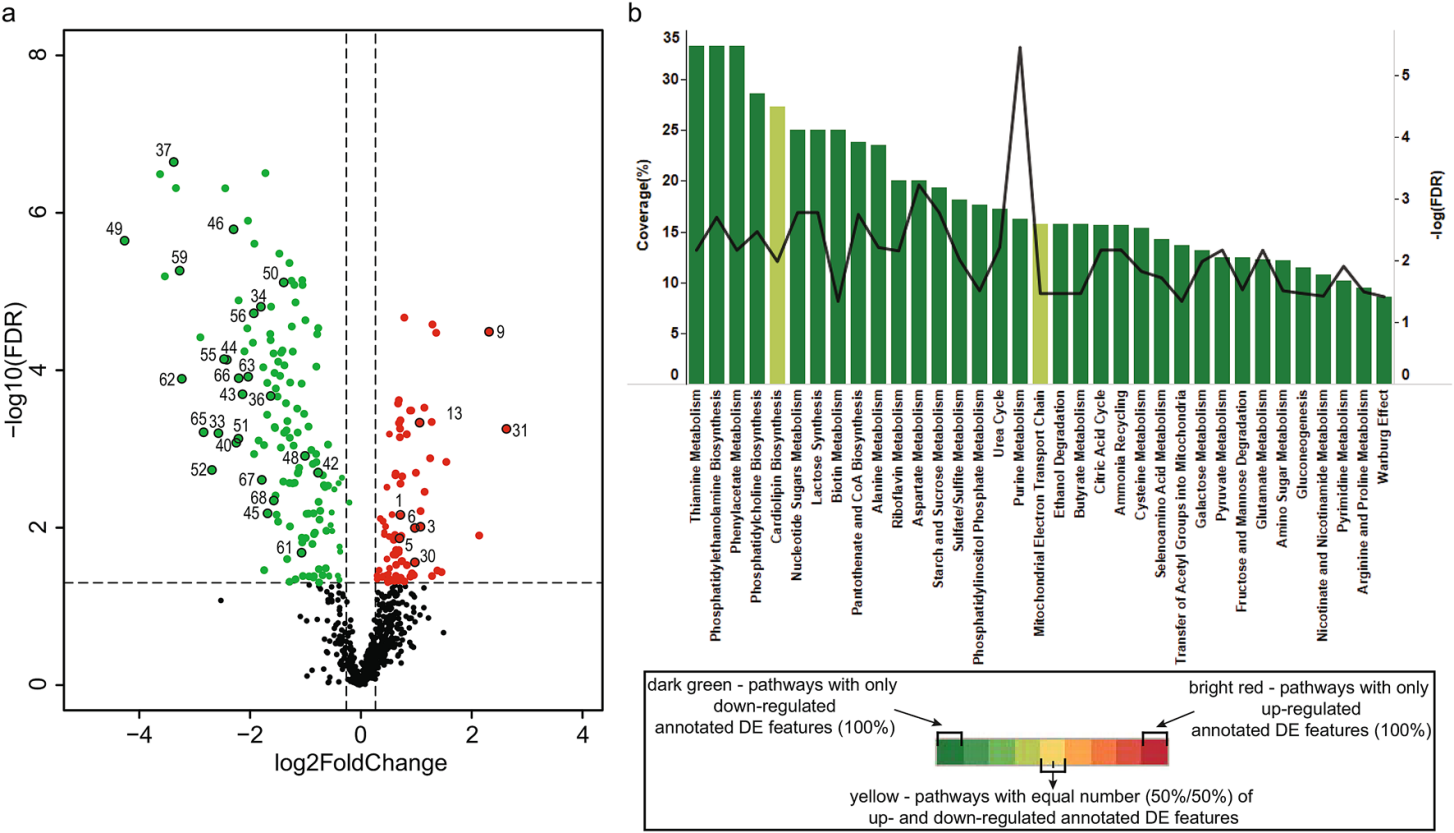
Exploratory analysis of data at 15 min of ischemia. (**a**) Volcano plot showed the profile of changes in the myocardial tissue metabolome at 15 min. The significantly altered features were colour coded respecting the selection threshold of FDR < 0.05 (horizontal dashed line); green and red dots represent significantly down- and up-regulated features, respectively. Statistical calculations were done using MetaboAnalyst software. Annotated features are highlighted by numbers, which correspond to their name in Supplementary Tables S5 and S6 online. (**b**) Functional enrichment analysis of the annotated features represented by bar graph showed several significantly perturbed KEGG pathways. Pathway enrichment analysis was performed with MetaboAnalyst online tool (Fisher’s exact test, Q-value < 0.05 for FDR correction) and only pathway enriched by at least two input metabolites were considered. The colour gradient (green to red) represents the enrichment with down- and up-regulated annotated DE features: dark green—the pathways containing exclusively down-regulated annotated DE features, bright red—the pathways containing exclusively up-regulated annotated DE features, and all other in-between colours are pathways containing both down- and up-regulated features. Coverage (%) represents the relative level of functional sets to the enriched set defined in the tissue metabolome of early stage ischemia.

**Figure 5:**
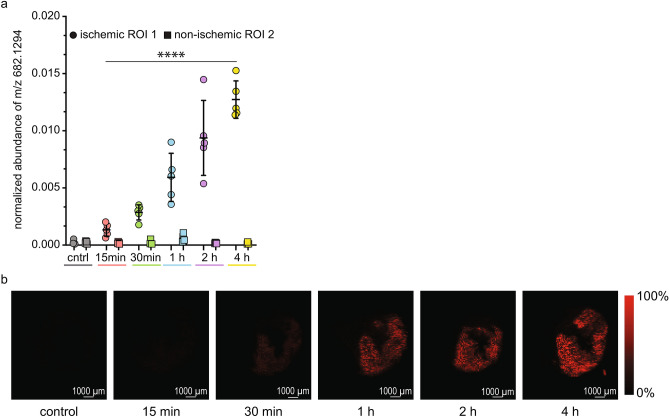
Possibly S-NADHX (m/z 682.1294) is nearly absent in the non-ischemic control but is significantly upregulated starting 15 min in the predicted ischemic area. (**a**) Temporal expression of possibly S-NADHX in ischemic (ROI 1, represented by circles) and non-ischemic (ROI 2, represented by squares) regions; n = 5 per time point (biological replicates); statistical significance was determined by multivariate regression analysis using Benjamini-Hochberg’s FDR (****P < 0.0005). (**b**) Image reconstruction of possibly S-NADHX in selected samples at the different time points; n = 5 per time point; abundance is represented by colour: black and red corresponding to low and high abundance, respectively.

**Figure 6:**
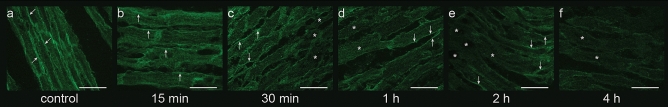
Immunofluorescence for Na_v_1.5. (**a**) Expression of Na_v_1.5 is evident at the plasma membrane and intercalated disks in the control heart; a fainter cytoplasmic signal was also observed. (**b**) Expression at 15 min of ischemia is similar to control. (**c**–**f**) Starting 30 min of ischemia, a patchy fainter expression was observed in the predicted ischemic region; asterisks (*) mark some of the cells and/or parts of cells with fainter signal at the plasma membrane and arrows indicate some of the cells with evident signal at the plasma membrane. The areas with fainter expression were more pronounced with prolonged ischemia with the strongest difference at 4 h; scale bar is 25 µm; n = 3 per time point (biological replicates).

The initial hour is the most important in post-mortem context because this period still lacks specific immunohistochemical markers. Already at 15 min, many features were up- and down-regulated (Fig. 4a, Supplementary Table S5 online). Pathway enrichment analysis was used to investigate the possible biological function of this very early differential expression. The 3 most highly covered pathways were thiamine metabolism, phosphatidylethanolamine biosynthesis, and phenylacetate metabolism (Fig. 4b). During the initial hour (15 min, 30 min, and 1 h), 32 and 39 annotated features were up- and down-regulated, respectively (Supplementary Table S6 online). The biggest significant fold change at 15 min was observed for m/z 682.1294—possibly corresponding to S-NADHX, which is a damage product of NADH. The expression of m/z 682.1294 was further increased with prolonged ischemia and its signal localized to the predicted ischemic area (Fig. 5). The expression of m/z 664.1188 (possibly corresponding to NADH) was upregulated starting at 2 h. Recently, NAD^+^/NADH ratio and increased NADH were reported to play a role in regulating localization of the voltage-gated sodium channel (Na_v_1.5) at the plasma membrane and single channel conductance^29,30^. Hence, we hypothesized that expression of Na_v_1.5 at the plasma membrane would be decreased with prolonged ischemia and tested it by immunofluorescence. As expected, the control non-ischemic hearts showed evident expression of Na_v_1.5 at the plasma membrane and intercalated disks as well as a fainter cytoplasmic signal (Fig. 6a). At 15 min of ischemia, the expression of Na_v_1.5 was similar to controls (Fig. 6b). Starting 30 min of ischemia, patches with fainter expression at the plasma membrane were seen in the predicted ischemic region (Fig. 6c). Prolonged ischemia led to a more pronounced decrease in localization at the plasma membrane and more extensive areas with decreased expression (Figs. 6d–f). The change became the most pronounced by 4 h.

## Discussion

The challenge in post-mortem diagnostics of EMI is the nearly normal appearance of myocardium by standard histology and the lack of specific and sensitive markers, especially during the initial hour of ischemia^4^. Identification of markers can be accelerated by characterization of molecular changes in ischemic myocardium. This study is the first untargeted profiling by MALDI IMS to investigate metabolomic changes in rat myocardium specifically in context of EMI and directly from tissue section. MALDI IMS served as a good tool for evaluating and localizing ischemic area because it characterized multiple metabolic changes simultaneously and detected region-specific molecular changes in tissue that otherwise appeared normal by standard histology (e.g. 15 and 30 min of ischemia). Moreover, availability of ischemic myocardial metabolome could contribute to describing the molecular mechanisms and further assist in interpreting circulating biomarkers.

Measurement of multiple markers instead of a single marker was suggested for improving EMI diagnostics and can be performed using untargeted methods^4^. In previous profiling studies, the molecular changes were evaluated in lysed ischemic myocardium that was separated manually by cutting the tissue, which is difficult to do accurately in small rodents^15,31^. In our study, untargeted screening not only tested multiple molecules simultaneously but it also measured them directly from tissue section, thereby avoiding manual isolation of ischemic areas. In addition, the use of MALDI IMS allowed more precise localization of ischemic region, which was successfully done at all time points by analysing metabolomics dataset using unsupervised clustering (an unbiased statistical approach). Segregation of ischemic region was possible only because the sampling was done directly from tissue and the spatial position of molecules was retained during data acquisition, which is one of the strengths of MALDI IMS. Until now, MALDI IMS was used to characterize responses in diseases with evident tissue pathology (e.g. cancer and others)^5,26^. Our finding confirms that MALDI IMS can detect molecular perturbations even in absence of evident histological lesions (ischemic period ≤ 30 min). The similarity between locations of ischemic clusters and Cx43-positive areas further supports that unsupervised clustering resulted in accurate classification. Although MALDI IMS is a promising approach for post-mortem diagnostics of EMI, some additional research needs to be done before it can be applied in routine practice. Firstly, our study used an established ex-vivo rat model in order to produce robust and reproducible results and to know precisely the duration of ischemia. However, validation in human myocardium would need to be performed because some differences exist between rat and human (e.g. cardiac hemodynamics, electrophysiology, and heart rate). Also, little information is available about post-mortem stability of myocardial metabolites. Some metabolites could be less prone to degradation than others (e.g. lipids *vs*. nucleic acids) but it remains to be tested experimentally. Lastly, metabolomic profiling was performed on frozen tissue in order to avoid losing or displacing metabolites during fixation procedures. But formalin-fixed paraffin embedded tissues (FFPE) is the standard method for tissue handling in post-mortem pathology. Other investigators have evaluated the application of MALDI IMS on FFPE^28^ but our preliminary testing of rat FFPE samples resulted in low signal intensity (data not shown) most likely as a result of our experimental conditions (e.g. section thickness, the selected matrix, and the specifics of the mass spectrometer). Additional work is needed to clarify the type of metabolites that are best preserved in FFPE tissues for assessment by MALDI IMS.

Metabolic changes are the earliest responses to ischemia in myocardium. They occur as early as few minutes (e.g. depletion of high-energy phosphates in the form of creatine phosphate) and continue to change with prolonged interruption of blood flow^7^. Our study performed the first untargeted metabolomic screening of myocardium specifically during the initial 4 h of ischemia. Majority of other studies investigated metabolomic changes in biofluids. The only other global metabolomic profiling of myocardium during EMI was done at 1 h (also 1 and 10 days) but using lysed tissue and with focus on polar and lipid molecules^15,32^. Our metabolic screening was focused on molecules that could be detected in the negative polarity and desorbed with 9-aminoacridine (matrix of choice for imaging of metabolites) and included small nucleotides, sugars, and phosphorylated metabolites^28^. Our data is consistent with previous findings because significant metabolic changes in ischemic myocardium were observed starting at 15 min and continued to change with longer ischemia. Also, several previously reported metabolomic changes were confirmed. For example, ATP is utilized much faster than it is produced during ischemia and our results showed a decrease in a molecule with m/z 505.9898 (possibly corresponding to ATP) starting 15 min along with molecules possibly corresponding to other nucleotides (m/z 426.0235—possibly ADP, m/z 346.0585—possibly AMP, m/z 521.9847—possibly GTP, m/z 442.0184—possibly GDP, etc.)^7^. In addition, our metabolic screening discovered annotated DE features that have not yet been implicated in ischemic response. Importantly, additional work is needed to further validate the exact assignment of annotated features to a given metabolite by supplementary methods. Unfortunately, the chemical diversity among metabolites (e.g. size, polarities, type of molecule, and concentration) makes it challenging to detect all metabolites with one single approach. Due to the selected method in our study, certain changes could not be replicated. Lactate accumulates in response to ischemia but its m/z is 89.0244 (M–H), which is too small to be detected within the range that was selected in our study (m/z 100–2000). Creatine was also not detected most likely due to low desorption by the selected matrix. It was previously successfully measured in the positive polarity with a matrix called 2,5-dihydroxybenzoic acid^21^. MALDI IMS can be combined with diverse matrices to target various compounds (e.g. lipids, peptides, nucleotides)^14,33^. Performing such characterization would provide a more comprehensive metabolomic profile of ischemic myocardium. But the biggest challenge in the field of metabolomics remains incomplete annotation of molecules^5^. In our data, many features could not be matched to a known metabolite as evident from the number of annotated and non-annotated features. Similar results were obtained by a study that characterized metabolome of a healthy mouse heart: many detected features remained unidentified^10^. The development and increased annotations in the database called METASPACE (more IMS-centric database) is likely to improve annotations (https://metaspace2020.eu)^34^. Comparing annotation results obtained with HMDB vs. METASPACE would be of interest. Lastly, MALDI IMS does not allow absolute quantification of metabolites like other metabolic methods. Ideally, a good internal control should be developed in the future. In our work, this aspect was addressed by performing relative quantification between controls and ischemic samples.

**Figure 7:**
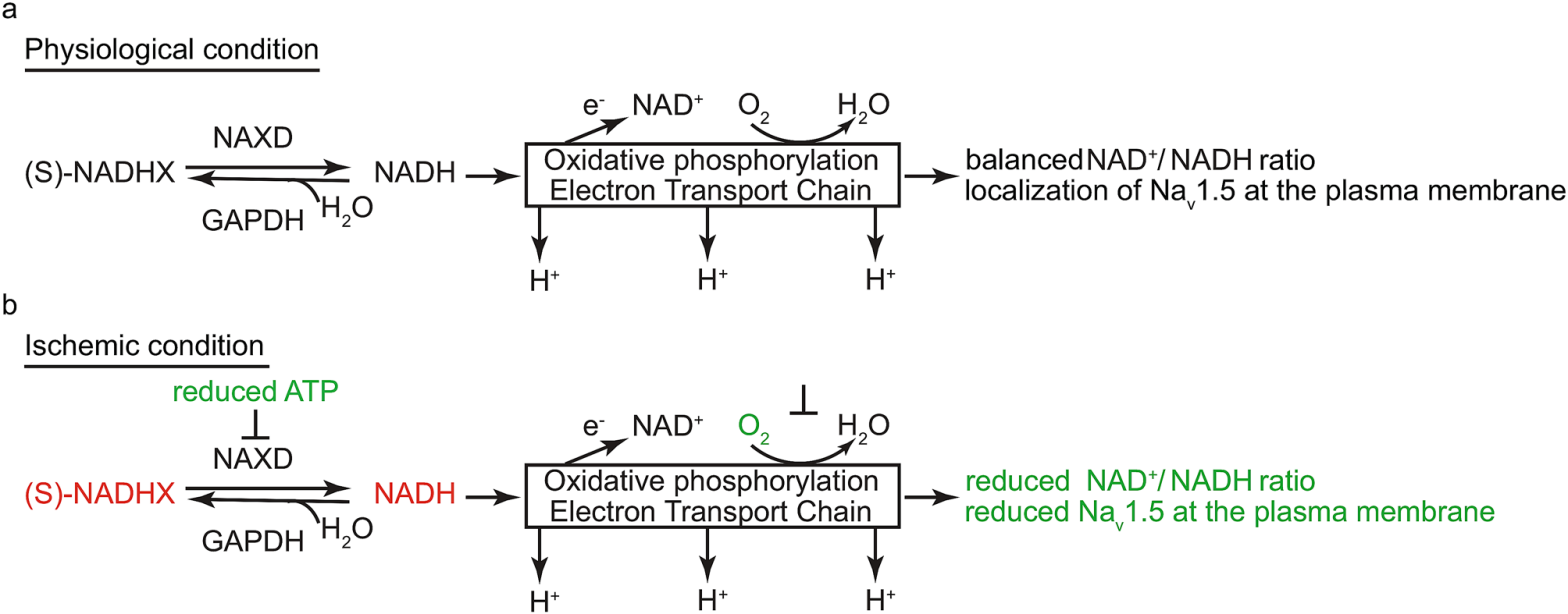
Schematic diagram of modifications in NAD^+^/NADH metabolism and localization of Na_v_1.5. (**a**) During physiological conditions, NAD^+^/NADH ratio is maintained, S-NADHX can be converted back into NADH by ATP-dependent NAD(P)HX dehydratase (NAXD), and Na_v_1.5 shows normal localization. (**b**) During ischemia, lack of oxygen affects oxidation of NADH to NAD^+^, thereby modifying NAD^+^/NADH ratio, reduced ATP is likely influencing conversion of S-NADHX back to NADH leading to its accumulation, and levels of Na_v_1.5 at the plasma membrane is reduced.

Metabolomics is used not only for biomarker discovery but also for understanding mechanisms leading to pathogenesis^15,32^. In this study, the upregulated annotated DE features were more interesting because the decreased features could be simply leaking from cells with damaged plasma membrane and, therefore, would be less specific. The molecule with m/z 682.1294 (possibly S-NADHX—the damage product of NADH) had the biggest fold change increase at 15 min. Under normal conditions, mitochondrial NADH is oxidized to NAD^+^ by donating electrons to the electron transport chain during oxidative phosphorylation^29,35^. Lack of oxygen during ischemia most likely leads to conversion of NADH into its damaged product S-NADHX instead of replenishing NAD^+^ pool. S-NADHX can be converted back into NADH by ATP-dependent NAD(P)HX dehydratase (NAXD also known as CARKD)^36,37^. However, reduced ATP during ischemia is likely to block this conversion, which may explain accumulation of S-NADHX (see schematic illustration in Fig. 7). Importantly, NAD^+^/NADH ratio is reduced during ischemia^29^ and is known to influence localization of Na_v_1.5 via modulation of deacelytation at lysine residue K1479 by sirtuin (SIRT)1 or phosphorylation at residue S1503 by protein kinase C (PKC)^29^. Based on this previous knowledge, the localization of Na_v_1.5 was evaluated by immunofluorescence and was found reduced at the plasma membrane with prolonged ischemia. The precise fate of Na_v_1.5 (whether it becomes degraded or re-distributed within the cardiomyocyte) remains to be investigated. Na_v_1.5 is a transmembrane channel and a predominant Na_v_ isoform in cardiomyocytes. It is responsible for rapid influx of sodium across cell membrane^30^. Highly coordinated movement of sodium and other ions (potassium and calcium) via various channels is essential for generation and propagation of cardiac action potential and subsequent excitation–contraction coupling^38^. Na_v_1.5 is involved in the depolarization phase of action potential. Until now, the role of Na_v_1.5 (encoded by *SCN5A* gene) has been implicated primarily in inherited cardiac arrhythmias, where the channel’s function is affected by certain inherited genetic variants^30^. Data in this study demonstrated that early ischemia alone can also influence Na_v_1.5, which together with other changes may subsequently lead to arrhythmia.

Although m/z 682.1294 (possibly S-NADHX) was selected as a follow-up annotated feature, other annotated DE features could be equally interesting. Several up-regulated DE features in our dataset matched to species involved in glycerolipid and glycerophospholipid metabolic pathways such as glycerol 3-phosphate, glycerophospholipids (phosphatidyl-choline, phosphatidyl-ethanolamine, phosphatidyl-glycerol, phosphatidyl-serine, phosphatidyl-inositol, phosphatidic acid), cyclic phosphatidic acid, lysophospholipids (lysoPI, lysoPE, lysoPC), and fatty acids (oleic, palmitic, and stearic acids). Although the majority of them were upregulated starting either 2 h or 4 h, a few were also increased during the initial hour. Although additional validation would be needed, this result is consistent with previously reported upregulation of several species of glycerophospholipids, lysophospholipids, and fatty acids at 1 h^15,21^. These changes most likely represent reorganization or break down of plasma membrane. Especially phosphatidyl-inositol has been proposed to contribute to the ischemia-induced irreversible cell injury^39^. Some by-products of glycerophospholipid degradation can also act as important signalling molecules or be used for synthesis of other metabolites. The species of these compounds in our data vary from the previously published work most likely as a result of using different methodology. The precise role of different species remains to be investigated. Also, further validation of the annotated features would be needed as well as additional verification of the involved pathways. Nonetheless, together these data support that metabolomics can be used to better understand molecular mechanisms that take place during ischemia. Combining metabolomics with other untargeted omics approaches (transcriptomics and proteomics) would be even more powerful for deciphering the pathophysiological mechanisms of EMI.

Understanding molecular changes in ischemic myocardium could also assist in interpreting circulating biomarkers, differentiating salvageable and irreversibly damaged myocardium, and even detecting potential drug targets for therapy. Metabolomic approach is already being used to investigate clinically relevant circulating biomarkers (e.g. serum, plasma, or blood) for identification of patients with EMI, differentiation of patients with unstable angina and ST-elevation MI, or risk stratification^40–42^. Metabolic changes in circulation can be a result of passive leakage of molecules from myocardial cell with damaged membranes, active release from surviving myocardium, release from circulating blood cells, or secretion by extra-cardiac tissue. For example, changes in taurine and free fatty acids in circulation have been associated with MI. While taurine was suggested to be a marker of disturbed cell integrity, circulating free fatty acids were proposed to be a consequence of increased lipolysis in adipose tissue^40^. Our data as well as results of other investigations suggest that at least some species of fatty acids may also originate from myocardium.

## Conclusion

Molecular changes were characterized during EMI using an established ex-vivo model and significant changes were detected as early as 15 min of ischemia. The DE molecules contained metabolites that were previously known to influence ischemia as well as new annotated and non-annotated features. MALDI IMS served as a good tool not only because it can measure hundreds of molecules simultaneously and directly from tissue section but also because it retains the spatial distribution of the molecules, which allows detecting region-specific changes and evaluating molecule’s localization by image reconstruction. Moreover, localization of ischemic areas by unsupervised clustering incorporates data from multiple markers. Characterization of metabolic changes specifically in myocardium provides a promising possibility to identify potential markers for post-mortem diagnostics, improve understanding of molecular mechanisms during EMI and MI, and interpret the meaning of circulating biomarkers.

## Methods

Detailed methods are available in the Supplementary material online.

### Animals and ex-vivo Langendorff heart perfusion

All animal maintenance, handling, experiments, and tissue collection were approved by the Swiss veterinary authorities, the local authorities at the University of Geneva (protocol number GE/83/16) and the procedures conformed to the guidelines from Directive 2010/63/EU of the European Parliament). All experiments were performed in accordance with these regulations and guidelines. Lewis male rats (195–275 g) were randomly assigned into control or ischemic groups. The rats were premedicated by subcutaneous injection of buprenorphine (0.05 mg/kg, Temgesic, Reckitt Benckiser AG, Switzerland). After 20 min, the rats were deeply anesthetized by one intraperitoneal injection of ketamine and diazepamum mix (100 mg/kg, Ketasol, Graeub AG, Switzerland and 5 mg/kg, Valium, Roche Pharma AG, Switzerland, respectively). This medication was chosen to minimize the adverse cardiovascular effect of anaesthesia. After confirmation of deep anaesthesia by absence of reflex in the posterior paws, the hearts were rapidly isolated, cannulated to the ex-vivo Langendorff system, and retrogradely perfused with oxygenated Krebs–Henseleit buffer (Supplementary Fig. S1a online). Euthanasia was performed by rapid excision of the heart under deep anaesthesia. The hearts were stabilized for 20 min, exposed to local permanent ischemia for 15 min, 30 min, 1 h, 2 h, and 4 h (n = 5 per time point: 5 biological replicates at each time point, 30 samples in total) by ligation of LAD using silk suture (6–0 Perma Hand, BV-1, ETHICON), and subsequently immediately snap frozen. The use of optimal cutting temperature (OCT) polymer was intentionally omitted because OCT can lead to analyte ion suppression in MS experiments. Control hearts were subjected to the same procedure, except the suture was only passed under LAD but not tied, and were maintained by Langendorff apparatus for 1 h after stabilization for 20 min. Success of LAD ligation was confirmed by Evans blue and triphenyltetrazolium chloride, H&E, immunohistochemistry for connexin43 (Cx43), and by monitoring the changes in the left ventricular developed pressure (LVDP) and coronary flow (Supplementary Figs. S2–S5 and Table S1 online).

### Tissue preparation for MALDI IMS

Tissue cryosections (12 µm) were cut along the transverse plane and thawed onto pre-chilled SuperFrost Plus microscope slides (Thermo Scientific, USA) (Supplementary Fig. S1b online). Metabolites were desorbed from tissues by depositing 8 layers of 9-aminoacridine (Sigma-Aldrich, 10 mg/ml in 70:30 MeOH:H_2_O) using an automated SunCollect spraying device (SunChrom, Friedrichsdorf, Germany) at the following setting: 10 µl/min (layer 1), 20 µl/min (layer 2), 30 µl/min (layer 3), 40 µl/min (layers 4–8) and spray height of 25.32 mm in z axis^28^.

### MALDI IMS instrumentation and data acquisition

Tissue imaging was performed using a MALDI-LTQ-Orbitrap XL equipped with a 337 nm N2 laser operating at 60 Hz (Thermo Scientific, Bremen, Germany); laser spot size 50 μm. Analysis were performed in negative polarity mode with a mass range from 100 to 2000 m/z at a resolution of 60,000, while automatic spectral filtering (ASF) and automatic gain control (AGC) were switched off. Fifteen laser shots and laser energy of 18 µJ were set to obtain the best S/N ratio. MALDI plate motion was fixed with a raster step size of 100 µm at 1 µscan/step. The mass calibration of the Orbitrap analyser alone was checked once a day with external calibrant to ensure operation within the < 10 ppm instrument specifications. Analyses of samples were randomized over different days. MS/MS fragmentation profile was confirmed for several selected annotated features (few MS/MS are shown in Supplementary Fig. S6).

### MALDI data processing and multivariate statistical analysis

Centroid data from TI.raw files were exported into imzML and idb files using ImageQuest software (Thermo Fisher Scientific, version 1.1.0). The imzML files were opened with MSiReader (v1.00, MatLab R2012b) and intensity data from a region of interest (ROI) were extracted into excel file using ± 20 ppm and a pre-defined list of m/z values, which was obtained by peak-picking from the entire imzML files using R packages (MALDIquant and MALDIquantForeign, R version 3.4.1)^43^ with the following specifications: mass tolerance of 0.001, minFrequency = 0.5, and nb = 100 (R code for peak-picking is available upon request). Duplicate peaks were removed by averaging neighbouring m/z values within ± 20 ppm range to adjust for possible mass shifts. The extracted data from ROI was further processed to extract meaningful peaks for subsequent multivariate analysis. Peaks fulfilling the following criteria were retained: 1) peak was present in more than 10% of pixels; 2) peak was found in at least 3 samples in one treatment group; and 3) its abundance exceeded 20,000. The final list contained 794 m/z features (Supplementary Table S2 online). Multivariate analyses were done from 2 ROIs (n = 5 per each time point): ROI 1 was in the area of the left ventricular wall predicted to be ischemic and ROI 2 was in septum (presumed non-ischemic area) (Supplementary Fig. S1c online). For both ROI 1 and 2, the statistical comparison was done between ROIs from controls (samples without ischemia) *vs.* ROIs from ischemic samples.

Data were normalized and transformed by applying MS-total useful signal (MSTUS)^44^ and log2, respectively. Multivariate regression analysis was done in R (version 3.5) using Limma package (version 3.8)^45^. Significantly differentially expressed (DE) features were identified using false discovery rate (FDR) < 0.05 (Benjamini–Hochberg adjusted) and matched to a custom list of known metabolites using ± 10 ppm range. The custom list was compiled and is constantly updated by our lab from the following sources: Human Metabolome Database (HMDB, http://www.hmdb.ca/), Lipidmaps (www.lipidmaps.org), Metlin (http://metlin.scripps.edu/index.php) and mzcloud (www.mzcloud.org). The profile of DE features at 15 min was demonstrated by volcano plot using “ggplot2” package in R: the DE features for the volcano plot were identified using MetaboAnalyst online tool (http://www.metaboanalyst.ca/, FDR < 0.05, fold change = 1.5). Pathway enrichment analysis was performed with MetaboAnalyst online tool (Fisher’s exact test, Q-value < 0.05 for FDR correction) and Kyoto Encyclopedia of Genes and Genomes (KEGG) database.

### Cluster analysis and image visualization

Clustering was performed in the R (version 3.4.1) using MALDIquant (version 1.16.4), MALDIquantForeign (version 0.11), and Cardinal (version 1.8.0) packages^23,43^. Data was segmented using spatially-aware K-means clustering (r = 1, k = 6). Individual ion distributions were visualized by image reconstruction either in R environment or using MSiReader (version 1.00, MatLab R2012b) normalized over TIC with ± 20 ppm (Supplementary material online, Supplementary Fig. S1c online).

### Immunohistochemistry and immunofluorescence

For immunohistochemistry, cryosections (12 μm) were fixed in pre-chilled methanol (5 min), permeabilized, blocked, probed with primary antibody recognizing multiple phospho-forms of Cx43 (rabbit polyclonal, Alpha Diagnostic Intl. Inc. Cx43B12-A, 1:100), incubated with biotinylated secondary antibody (anti-rabbit, Vector Laboratories, 1:200) and streptavidin-HRP (1:300), visualized with colorimetric detection kit (Vector AEC, Vector Laboratories), and counterstained with hematoxylin. For immunofluorescence, cryosections (7 μm) were dried, fixed, blocked, permeabilized, incubated with primary antibody for Na_v_1.5 (custom-made rabbit polyclonal for amino acids 493–511 of rat Na_v_1.5, gift from Prof. Hugues Abriel^46^, 1:200) and secondary DyLight 488 antibody (goat anti-rabbit IgG H&L, Abcam, 1:200). For both IHC and IF, negative controls (the primary antibody was omitted and replaced with incubation buffer) showed negative results.

## Supplementary Information

Supplementary Information

## Data Availability

The data is provided in the Supplementary Material Online. The data is also available in METASPACE.
