# Supplementary material for: Detecting early myocardial ischemia in rat heart by MALDI imaging mass spectrometry
Source: Sci Rep. 2021 Mar 4;11:5135. doi: 10.1038/s41598-021-84523-z (PMC7933419; doi:10.1038/s41598-021-84523-z)
Supplement: Supplementary file 1 — Supplementary Information [file 41598_2021_84523_MOESM1_ESM.pdf]

Detecting Early Myocardial Ischemia in Rat Heart by MALDI Imaging Mass Spectrometry

Online Supplementary Material for *Scientific Reports*

Aleksandra Aljakna Khan[1], Nasim Bararpour[2,3], Marie Gorka[4], Timothée Joye[2,3], Sandrine Morel[5], Christophe Montessuit[5], Silke Grabherr[1,2], Tony Fracasso[1], Marc Augsburger[1,2], Brenda R. Kwak[5], Aurélien Thomas[1,3], Sara Sabatasso[1,2*]

[1] University Centre of Legal Medicine, Lausanne-Geneva, Rue Michel-Servet 1, 1211 Geneva, Switzerland
[2] University Centre of Legal Medicine, Lausanne-Geneva, Rue Vulliette 04, 1000 Lausanne, Switzerland
[3] Faculty of Biology and Medicine, Lausanne University Hospital, University of Lausanne, Rue Vulliette 04, 1000 Lausanne, Switzerland
[4] Ecole des Sciences Criminelles/School of Criminal Justice, Faculty of Law, Criminal Justice, and Public Administration, University of Lausanne, 1015 Lausanne-Dorigny, Switzerland
[5] Department of Pathology and Immunology, University of Geneva, Rue Michel-Servet 1, 1211 Geneva, Switzerland

Corresponding author:

Sara Sabatasso, M.D.
University Centre of Legal Medicine Lausanne-Geneva (CURML)
Unit of Forensic Medicine, CMU
Rue Michel-Servet 1
1211 Geneva
Switzerland

Tel: +41 (0)79 556 9118
E-mail: sara.sabatasso@unige.ch

TABLE OF CONTENTS

SUPPLEMENTARY MATERIAL 2
Supplementary Methods 2
Animals and *ex-vivo* Langendorff heart perfusion 2
Immunohistochemistry and immunofluorescence 2
Confirmation of ischemia in the *ex-vivo* Langendorff model 3
Evans blue and TTC staining 3
H&E 3
Immunohistochemistry for dCx43 3
Changes in left ventricular developed pressure (LVDP) and coronary flow 4
Supplementary Figures 5
Figure S1 – diagram of the experimental design and workflow 5
Figure S2 – Evans blue and TTC staining 6
Figure S3 – H&E staining 7
Figure S4 – immunohistochemistry for dephosphorylated Cx43 (dCx43) 7
Figure S5 – LVDP and heart rate 8
Figure S6 – MS/MS 9
Supplementary Tables 10

SUPPLEMENTARY MATERIAL

Supplementary Methods

Animals and *ex-vivo* Langendorff heart perfusion

All animal experiments were performed according to the approved protocol by the Swiss veterinary authorities (GE/83/16). Lewis male rats (195-275 g) were housed in controlled, conventional conditions (20-24°C and 30-70% humidity) in an authorized facility under veterinary supervision with free access to food and water. All rats were allowed a minimum one-week adaptation period after arrival to the local animal facility and were randomly assigned into control or ischemic groups. The rats were premedicated by subcutaneous injection of buprenorphine (0.05 mg/kg, Temgesic, Reckitt Benckiser AG, Switzerland). After 20 minutes, the rats were deeply anesthetized by one intraperitoneal injection of ketamine and diazepamum mix (100 mg/kg, Ketasol, Graeub AG, Switzerland and 5 mg/kg, Valium, Roche Pharma AG, Switzerland, respectively). This anesthetic mix was chosen to minimize the adverse cardiovascular effect. After confirmation of deep anesthesia by absence of reflex in the posterior paws, the hearts were rapidly isolated, cannulated via the aorta to the *ex-vivo* Langendorff system, and retrogradely perfused under constant pressure at 37°C with oxygenated Krebs-Henseleit buffer solution (NaCl 118 mM, KCl 4.7 mM, $MgSO_4$ 1.19 mM, KH_2PO_4 1.2 mM, $CaCl_2$ 1.36 mM, $NaHCO_3$ 25 mM, Glucose 11 mM) (Fig. S1A). Euthanasia was performed by rapid excision of the heart under deep anesthesia. Throughout the experiments, the temperature was monitored. The left ventricular developed pressure (LVDP) and heart rate were measured via a balloon, which was inserted into the left ventricle, and connected by a pressure transducer to a computer with appropriate software (Lab Chart, ADInstruments). LVDP was fixed around 6 mmHg for all of the hearts at the beginning of the stabilization period. The hearts were stabilized for 20 min and local ischemia was induced by complete ligation of LAD using silk suture (6-0 Perma Hand, BV-1, ETHICON). Hearts were exposed to 15 min, 30 min, 1 h, 2 h, and 4 h ischemia (n = 5 per time point: 5 biological replicates at each time point, 30 samples in total) and were immediately frozen. The use of optimal cutting temperature (OCT) polymer was intentionally omitted because OCT can lead to analyte ion suppression. Control hearts were subjected to the same procedure, except the suture was not tied (it was only passed under LAD). After 20 min of stabilization, control hearts were maintained by Langendorff perfusion for 1h. Success of LAD ligation was confirmed by staining with Evans blue and triphenyltetrazolium chloride (TTC), H&E, immunohistochemistry for connexin43 (Cx43) as well as by monitoring the changes in LVDP and coronary flow (see below, Figs. S2-S5 and Table S1).

Immunohistochemistry and immunofluorescence

For immunohistochemistry, cryosections from frozen hearts (12 µm) were fixed for 5 min in pre-chilled methanol, permeabilized in 0.2% Triton X-100, neutralized with 0.5 M NH_4Cl in PBS, incubated with 3% H_2O_2, blocked with bovine serum albumin (BSA) and avidin/biotin blocking kit (Abcam), probed with primary antibody that recognizes multiple phospho-forms of Cx43 (rabbit polyclonal, Alpha Diagnostic Intl. Inc. Cx43B12-A, 1:100), incubated with biotinylated secondary antibody (anti-rabbit, Vector Laboratories, 1:200) and streptavidin-HRP (1:300), visualized with colorimetric detection kit (Vector AEC, Vector Laboratories), and counterstained with hematoxylin (Fig.1 p-y in the main text of the article). For immunofluorescence, cryosections (7 µm) were dried for 15 min at RT, fixed for 10 min in

pre-chilled acetone, blocked and permeabilized with blocking buffer (1% BSA, 10% goat serum, and 0.5% TritonX-100 in PBS), probed with primary antibody for $Na_V1.5$ (custom-made rabbit polyclonal for amino acids 493-511 of rat $Na_V1.5$, gift from Prof. Hugues Abriel, 1:200), and incubated with secondary DyLight 488 antibody (goat anti-rabbit IgG H&L, Abcam, 1:200). Coverslips were mounted with FluorSave (Merck Millipore) (Fig.6 in the main text of the article). Negative controls (the primary antibody was omitted and replaced with incubation buffer) were performed for both IHC and IF and showed negative results.

Confirmation of ischemia in the *ex-vivo* Langendorff model

Cessation of perfusion to the area below the ligature and ischemic changes were confirmed by four methods: 1) staining with Evans blue and triphenyltetrazolium chloride (TTC), 2) H&E staining, and 3) immunohistochemistry for dephosphorylated connexin 43 (dCx43), 4) monitoring change in left ventricular developed pressure (LVDP) and coronary flow.

Evans blue and TTC staining

While Evans blue dye demarcates the perfused area, the colourless TTC allows distinguishing between viable and non-viable myocardium in the area at risk. TTC gives a vivid red coloration to viable tissue but does not stain the non-viable myocardium, which remains pale (Fig. S2). This dual staining was applied to a heart exposed to 4 h of ischemia. At the end of the ischemic period, heart was perfused with Evans blue, briefly rinsed with Krebs-Henseleit buffer to remove the excess of dye, and snap frozen. On the next day, hearts were cut into transverse slices and incubated with TTC (0.25% in 0.1 M phosphate buffer) at 37°C for 20 min. Hearts were fixed in 10% formalin, rinsed in PBS, and photographed. Using this method, the presence of non-viable pale myocardium in *ex-vivo* ischemic rat heart was confirmed (Fig. S2).

H&E

The earliest histological, non-specific ischemic change is hypereosinophilia of cardiomyocytes, which can be detected by H&E staining. The H&E staining was applied to hearts exposed to ischemia for 1 h and 4 h. Briefly, hearts were removed from Langendorff model at the end of ischemic time, fixed for 2 h in 2% paraformaldehyde (PFA) on ice, incubated for 1 h in sucrose (10% in PBS), placed in 70 % ethanol, and subsequently embedded in paraffin using standard protocol. Sections (5 μm) were deparaffinized and stained with H&E. Hypereosinophilia of cardiomyocytes was detected at 1 h and 4 h and localized to the area, which was predicted to be ischemic (based on the location of the ligation) (Fig. S3).

Immunohistochemistry for dCx43

In a healthy heart, the gap junction protein Cx43 is present primarily at the intercalated discs but some Cx43 is also found in mitochondria. The location and function of Cx43 channels is influenced by various posttranslational modifications, which are also affected by ischemia. Initial 30 min of ischemia increases dephosphorylation of Cx43 at Ser368 (dCx43). Changes in dCx43 were assessed by immunohistochemistry using antibodies recognizing dCx43 in hearts exposed to 1 h and 4 h ischemia. Evident increased dCx43 was observed at both 1 h and 4 h and localized in the area, which was predicted to be ischemic (similar distribution of hypereosinophilic cardiomyocytes) (Fig. S4). Briefly, sections from PFA-fixed hearts (5 μm) were deparaffinized. Antigen retrieval was performed by microwaving for 15 min at 95°C in

Dako Target Retrieval Solution. Tissue sections were treated with 1% H_2O_2, blocked with BSA, probed with primary dCx43 antibody (mouse monoclonal, Invitrogen 13-8300, 1:500), incubated with biotinylated secondary antibody (anti-mouse, Vector Laboratories, 1:200) and streptavidin-HRP (1:300), visualized with colorimetric detection kit (Vector AEC, Vector Laboratories), and counterstained with hematoxylin.

Changes in left ventricular developed pressure (LVDP) and coronary flow

At the end of stabilization period (baseline), the LVDP and heart rate were comparable among the groups: no statistical difference was observed (Figs. S5A and S5B). Both LVDP and coronary flow were reduced after induction of ischemia by ligation of LAD (Table S1, Figs. S5C - D). Each sample showed reduction in LVDP (Fig. S5E). LVDP was calculated as following: LVDP = LVESP – LVEDP, where LVESP and LVEDP correspond to left ventricular end-systolic and end-diastolic pressures, respectively. Statistical significance was calculated using Wilcoxon t-test. Differences were considered statistically significant at values of $P<0.05$.

Supplementary Figures

Figure S1 – diagram of the experimental design and workflow

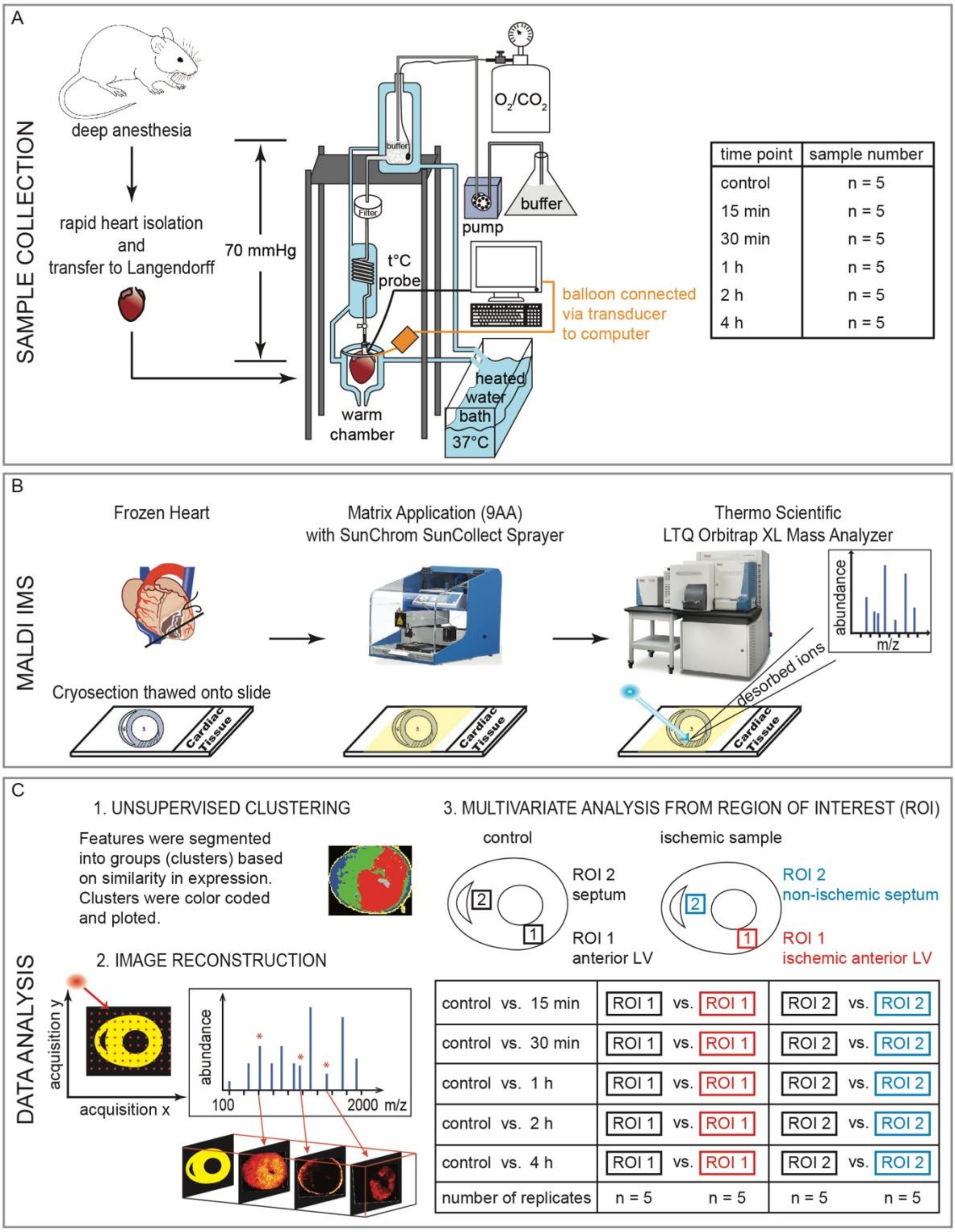

Figure S1. Experimental design and workflow of the study. (**A**) Sample preparation and *ex-vivo* Langendorff model; the image of a mouse was obtained from commons.wikimedia.org: "Vector diagram of laboratory mouse (black and white)" by Gwilz, CC Attribution-Share Alike 4.0. (**B**) MALDI IMS workflow: cryosections from hearts were cut on a transverse plane, thawed onto the slide, sprayed with 9-aminoacridine (9AA), and analysed by LTQ Orbitrap Mass Analyser; images of SunChrom SunCollect Sprayer and Thermo Scientific LTQ Orbitrap XL Mass Analyzer are reproduced from the technical manuals for the instruments. (**C**) Data was analysed by 3 different method: unsupervised clustering, image reconstruction, and multivariate analysis from 2 regions of interest.

Figure S2 – Evans blue and TTC staining

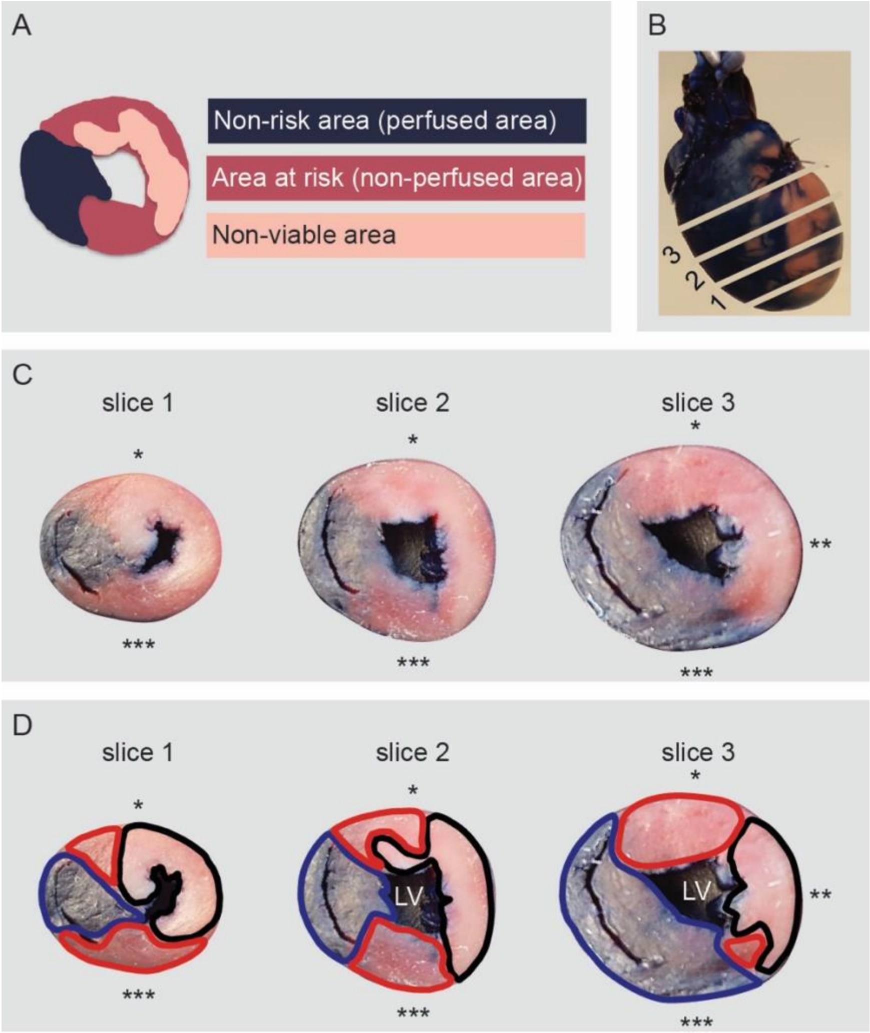

Figure S2. Evans blue and TTC staining confirmed successful coronary artery ligation. (**A**) Diagram of a heart's cross section explaining the colorations. (**B**) representative examples of a heart that was exposed to 4 h ischemia, perfused with Evans blue to demarcate perfused and non-perfused areas, and subsequently cut into several transverse slices (illustrated by lines) for further staining with TTC; slices 1-3 indicate the position in relation to the apex. (**C**) Representative examples of heart's slices stained with both Evans blue and TTC. (**D**) Same slices as in (C) but with demarcated areas: non-risk, perfused area of myocardium stained blue (outlined by blue lines); viable myocardium in the area at risk stained red by TTC (outlined by red lines); pale areas demarcated in black represent non-viable, pale myocardium. Slice 1 and slice 3 were closest and furthest from the apex, respectively. LV – left ventricle, * - anterior left ventricular wall, ** - lateral left ventricular wall, *** - posterior left ventricular wall.

Figure S3 – H&E staining

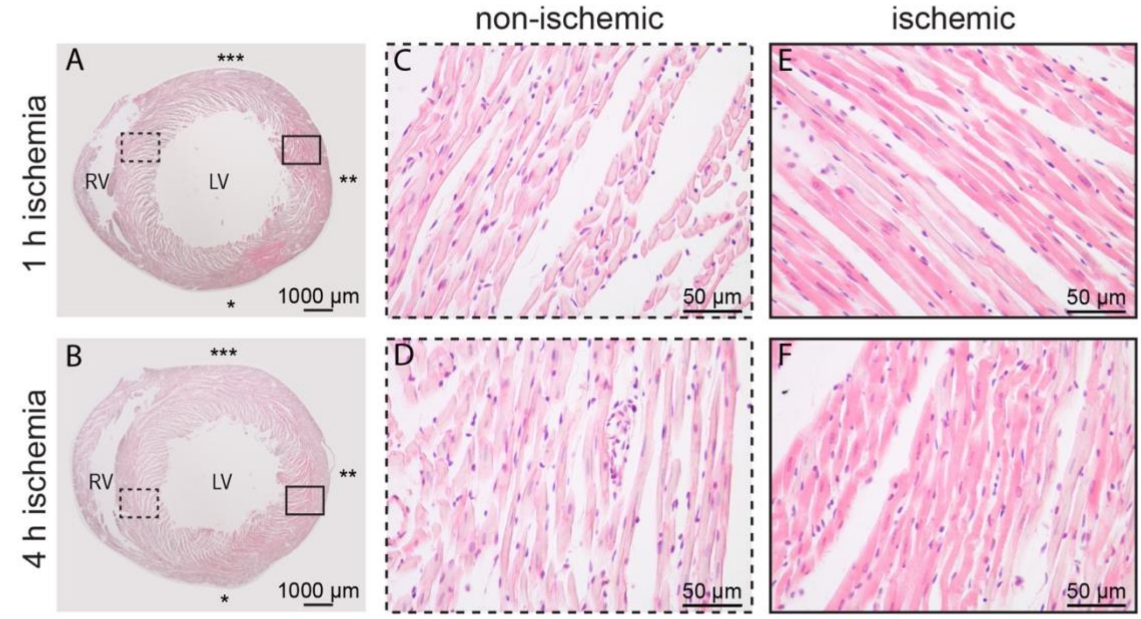

Figure S3. Cardiomyocyte hypereosinophilia (the earliest, non-specific, ischemic change that can be detected by H&E staining). (**A, B**) Overview of stained cross sections from hearts exposed to 1 h and 4 h ischemia; dashed line and black rectangles highlight non-ischemic and ischemic regions, respectively. These regions are shown at higher magnification in C-F. LV – left ventricle, RV – right ventricle, * - anterior left ventricular wall, ** - lateral left ventricular wall, *** - posterior left ventricular wall. (**C, D**) Non-ischemic septum, showing lack of cardiomyocyte hypereosinophilia. (**E, F**) Hypereosinophilic cardiomyocytes in the predicted ischemic area of the left ventricular wall.

Figure S4 – immunohistochemistry for dephosphorylated Cx43 (dCx43)

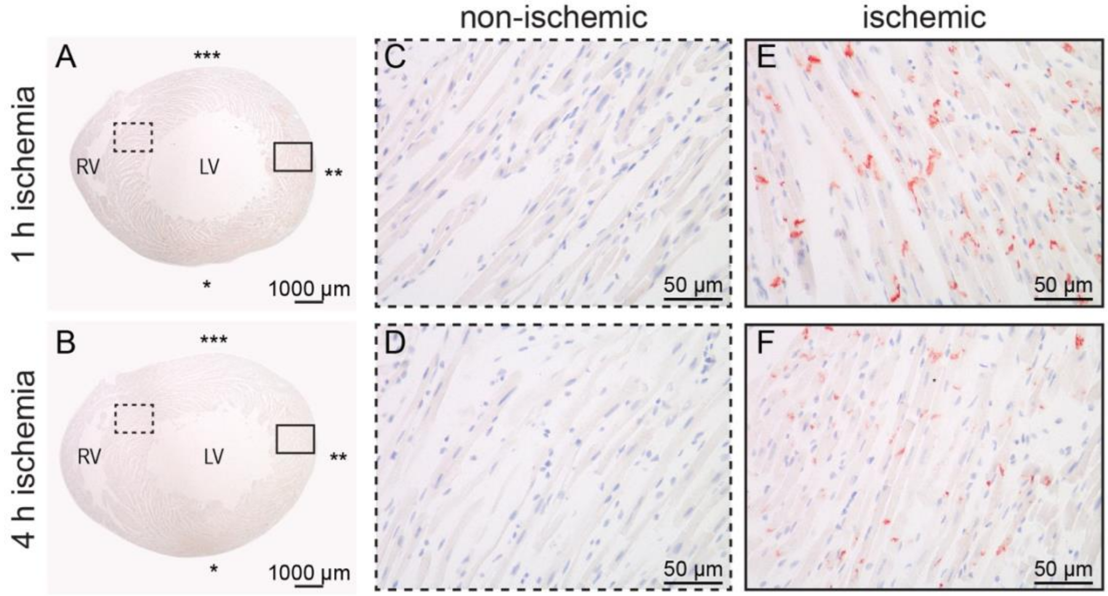

Figure S4. Dephosphorylated Cx43 (dCx43) is increased by ischemia. (**A, B**) Overview of stained cross sections from hearts exposed to 1 h and 4 h ischemia; dashed line and black rectangles highlight non-ischemic and ischemic regions, respectively. These regions are shown at higher magnification in (C-F). LV – left ventricle, RV – right ventricle, * - anterior left ventricular wall, ** - lateral left ventricular wall, *** - posterior left ventricular wall. (**C, D**) Non-ischemic septum showing no staining for dCx43. (**E, F**) Positive dCx43 staining at the intercalated discs in the predicted ischemic area of the LV wall. Antibody information is in the Supplementary methods.

Figure S5 – LVDP and heart rate

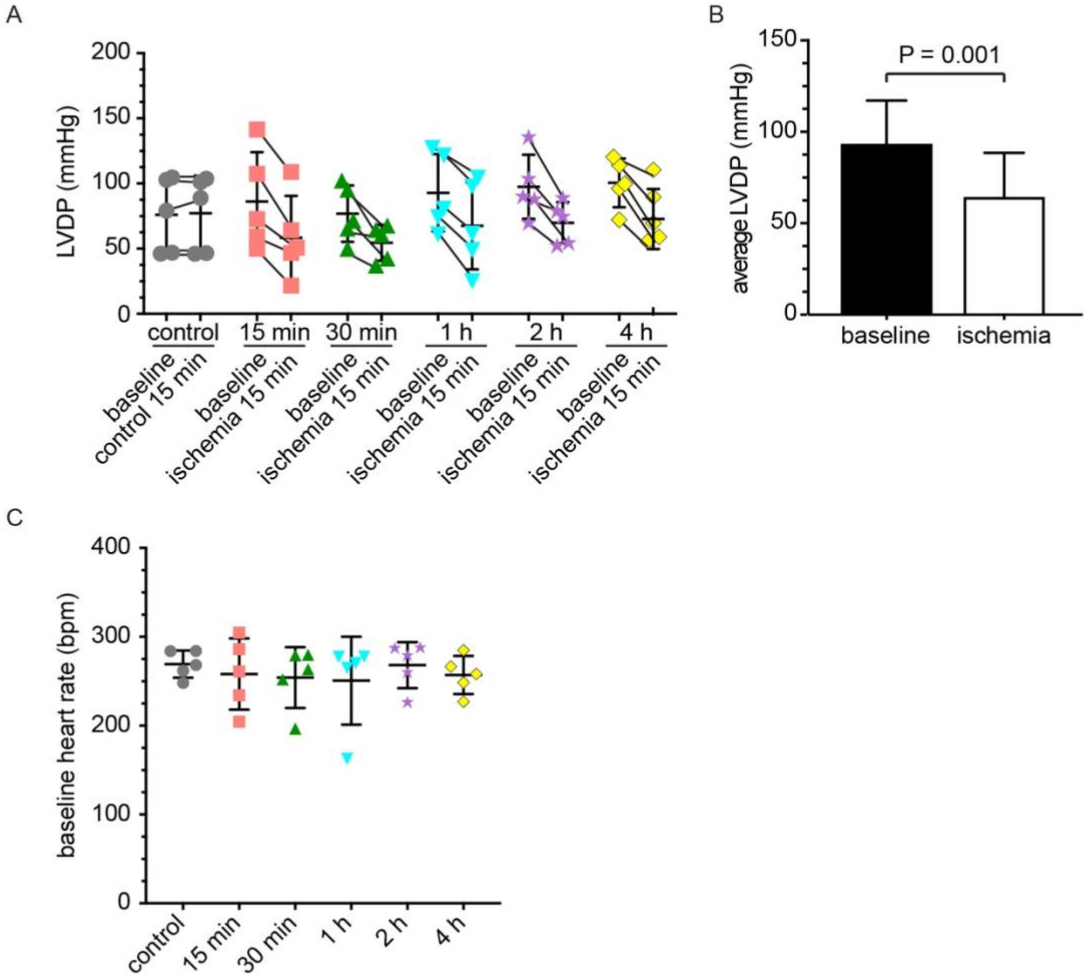

Figure S5. LVDP and heart rate in *ex-vivo* Langendorff rat hearts. (**A**) LVDP at baseline (the end of stabilization period) and 15 min after LAD ligation by group (n = 5 per group); controls -no ischemia: 15 min after the end of stabilization period. (**B**) Average LVDP in all of the ischemic samples (n=25) at baseline and 15 min after LAD ligation; statistical significance was calculated using Wilcoxon t-test. (**C**) Baseline heart rate (at the end of stabilization period) in each treatment group; n = 5 per group.

Figure S6 – MS/MS

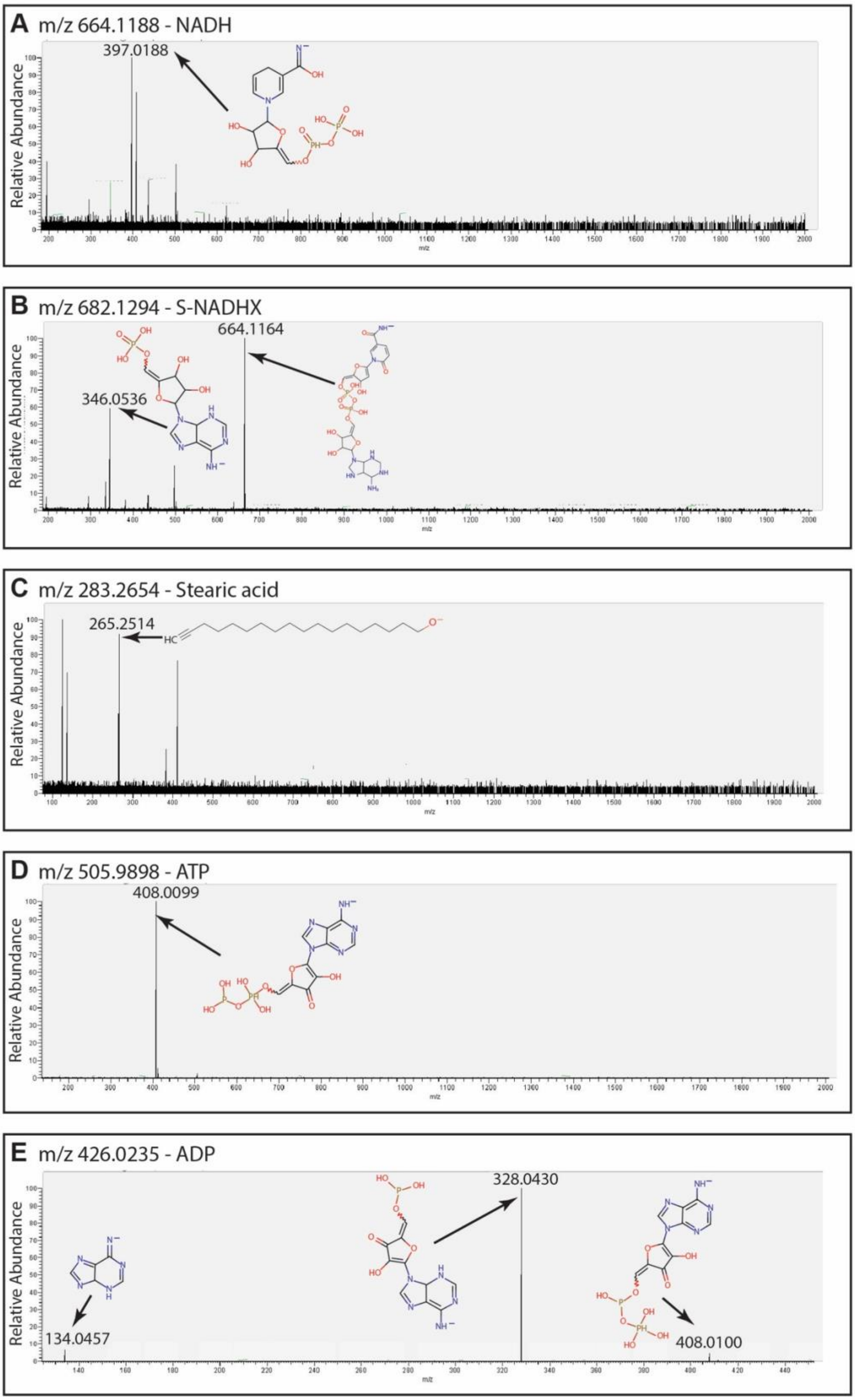

Figure S6. Fragmentation results (MS/MS spectra) of several annotated features acquired with MALDI-LTQ Orbitrap XL in negative ionization mode in rat heart. (**A**) m/z 664.1188, NADH, collision energy 30 (**B**) m/z 682.1294, S-NADHX, collision energy 30. (**C**) m/z 283.2654, stearic acid, collision energy 50. (**D**) m/z 505.9898, ATP, collision energy 30. (**E**) m/z 426.0235, ADP, collision energy 30.

Supplementary Tables

Supplementary Table S1. Mean LVDP and coronary flow at baseline (end of stabilization period) and after 15 min of ischemia as well as their percentage decrease; statistical significance was calculated by Wilcoxon t-test

	**Baseline** (mean ± SE) n=25	**Ischemia (15 min)** (mean ± SE) n=25	**% Decrease** (mean ± SE) n=25
LVDP (mmHg)	90.8 ± 5.3	64.6 ± 4.8*	29.4 ± 2.9
Cardiac flow (ml/30 sec)	4.6 ± 0.2	3.1 ± 0.9**	32.7 ± 1.8

* P=0.001 ** P<0.0001

Supplementary Table S2. List of all detected features in rat heart: 794 m/z values were detected using 9-aminoacridine and MALDI LTQ Orbitrap XL in negative polarity.

#	m/z	#	m/z	#	m/z	#	m/z	#	m/z	#	m/z
1	122.9767	55	191.0626	109	231.0438	163	273.0010	217	303.2341	271	329.2498
2	122.9863	56	192.0347	110	232.0296	164	273.0122	218	304.0621	272	329.2709
3	124.0083	57	192.9948	111	232.8845	165	273.0350	219	304.2375	273	330.2531
4	125.0077	58	193.0781	112	234.0452	166	274.0072	220	305.0245	274	330.9610
5	125.0117	59	194.0139	113	236.0243	167	274.0381	221	305.2497	275	331.2655
6	126.0041	60	194.0621	114	238.0402	168	274.9968	222	306.0565	276	331.9835
7	126.0126	61	194.0759	115	238.8928	169	276.0922	223	306.0777	277	332.2277
8	128.0363	62	194.0820	116	239.0355	170	277.2185	224	306.1392	278	334.1342
9	132.0312	63	195.0854	117	239.0837	171	278.1079	225	307.0071	279	335.0002
10	133.0152	64	195.9931	118	239.9258	172	279.0279	226	307.0811	280	335.0663
11	134.0482	65	196.0296	119	241.0131	173	279.2341	227	307.0962	281	336.0172
12	134.9863	66	196.0401	120	241.0629	174	280.0120	228	307.2654	282	336.9769
13	135.0323	67	197.1193	121	241.9308	175	280.2375	229	308.0569	283	337.0176
14	136.0084	68	197.9410	122	242.0164	176	280.9895	230	308.9844	284	338.9900
15	137.9877	69	198.9094	123	242.0350	177	281.2409	231	310.9715	285	339.9887
16	138.9812	70	198.9190	124	242.0466	178	281.2497	232	311.0001	286	340.9534
17	140.0129	71	199.0024	125	243.9579	179	282.2531	233	311.1711	287	341.0106
18	146.0469	72	199.1350	126	243.9673	180	283.0051	234	311.2239	288	342.9614
19	149.9877	73	199.9380	127	244.0273	181	283.2443	235	312.9508	289	344.0199
20	150.0241	74	202.0190	128	244.9612	182	283.2654	236	312.9583	290	344.0413
21	150.9813	75	204.0346	129	245.9537	183	284.0457	237	312.9947	291	345.0733
22	151.0273	76	206.0139	130	249.0232	184	284.2687	238	313.0157	292	346.0473
23	152.0034	77	206.0867	131	249.8812	185	284.9844	239	314.0190	293	346.0585
24	152.9969	78	207.0452	132	250.0402	186	285.2722	240	314.9740	294	347.0410
25	153.0067	79	208.0295	133	251.9630	187	285.9684	241	315.0127	295	347.0601
26	154.0001	80	208.9860	134	253.0511	188	285.9779	242	315.0498	296	348.0444
27	154.9198	81	209.0232	135	253.2184	189	286.0613	243	316.0638	297	348.0668
28	154.9293	82	209.0806	136	254.9464	190	286.9698	244	316.0773	298	348.2226
29	158.9265	83	209.9620	137	255.0422	191	286.9791	245	316.0845	299	349.0453
30	162.8402	84	211.0024	138	255.2341	192	287.0655	246	317.0572	300	350.1288
31	166.0190	85	211.0523	139	256.2374	193	287.9824	247	318.0070	301	350.1386
32	167.0223	86	211.1350	140	257.9736	194	288.9830	248	318.0929	302	352.1447
33	167.9983	87	214.0517	141	259.0236	195	290.0893	249	320.0529	303	352.9509
34	168.0148	88	214.9527	142	259.9319	196	290.9688	250	320.9794	304	353.0107
35	168.0442	89	215.1300	143	259.9414	197	290.9762	251	322.0505	305	353.0819
36	171.0074	90	216.9105	144	260.0974	198	293.0436	252	322.9791	306	354.0625
37	171.1037	91	216.9201	145	261.0175	199	293.2133	253	323.0001	307	355.0263
38	174.0241	92	218.0139	146	261.9298	200	294.0719	254	323.0925	308	355.9625
39	174.9813	93	220.0296	147	262.1130	201	294.1037	255	324.0332	309	357.9992
40	176.9370	94	222.0089	148	264.0421	202	294.9402	256	324.9794	310	360.2590
41	178.0190	95	222.0453	149	266.9739	203	295.0051	257	325.0157	311	360.9720
42	179.0126	96	223.0025	150	267.0773	204	295.2290	258	325.1092	312	361.0158
43	179.9983	97	223.1351	151	267.9370	205	296.9634	259	325.1853	313	361.0472
44	180.9084	98	223.9777	152	268.9896	206	296.9844	260	326.0440	314	361.9753
45	181.9671	99	224.0246	153	269.2498	207	297.0207	261	326.1053	315	362.0060
46	182.0139	100	224.0348	154	269.9736	208	297.0392	262	326.1119	316	362.0520
47	182.9704	101	224.0705	155	270.9995	209	298.0710	263	327.2341	317	365.0922
48	183.9642	102	225.9473	156	271.0052	210	298.9798	264	328.0463	318	366.0277
49	184.9867	103	225.9568	157	271.0148	211	299.0001	265	328.0597	319	369.0231
50	185.9642	104	226.9552	158	271.0245	212	300.0033	266	328.1211	320	370.1361
51	187.0430	105	229.0130	159	271.9528	213	300.0898	267	328.2375	321	372.1193
52	187.0987	106	229.0488	160	272.0084	214	300.9959	268	328.9639	322	373.9732
53	190.0191	107	229.0549	161	272.0900	215	301.0663	269	328.9897	323	375.2319
54	191.0209	108	230.0116	162	272.9582	216	302.9996	270	329.0508	324	376.0446

Supplementary Table S2. ***Continued from previous page***

#	m/z	#	m/z	#	m/z	#	m/z	#	m/z	#	m/z
325	376.2353	379	428.0276	433	479.2792	487	545.9729	541	618.4059	595	695.4670
326	376.9460	380	429.0118	434	480.2746	488	548.0410	542	619.2902	596	696.1086
327	379.0278	381	429.2061	435	480.3108	489	549.9537	543	619.4092	597	696.3567
328	382.1204	382	429.9948	436	481.3142	490	556.0501	544	620.0215	598	696.4704
329	382.1315	383	430.9980	437	482.9626	491	557.0817	545	622.3374	599	696.9504
330	384.0340	384	431.1170	438	483.9738	492	558.0658	546	627.1235	600	697.1124
331	384.9857	385	431.2248	439	487.9793	493	559.0693	547	628.0576	601	697.3585
332	384.9969	386	433.0581	440	489.2636	494	562.0372	548	628.1272	602	697.4832
333	385.1489	387	433.2374	441	490.0160	495	562.3433	549	629.3456	603	698.1243
334	386.0348	388	433.9759	442	493.0538	496	563.3463	550	633.1280	604	698.3615
335	387.1336	389	434.0093	443	494.0379	497	564.9986	551	637.0126	605	698.4863
336	388.0098	390	434.0617	444	494.0570	498	565.0509	552	638.0321	606	699.3663
337	389.0132	391	434.2407	445	497.3187	499	565.1406	553	640.2941	607	699.4986
338	390.0035	392	435.0540	446	498.0446	500	565.9277	554	642.0035	608	700.0801
339	390.9547	393	435.2530	447	499.0481	501	566.0518	555	644.1480	609	700.5017
340	391.2268	394	436.0840	448	499.9475	502	567.9553	556	646.4370	610	701.5142
341	392.2302	395	436.2867	449	500.2796	503	571.2903	557	647.3723	611	702.0979
342	393.2425	396	437.2686	450	504.1146	504	572.0816	558	651.9021	612	703.0257
343	397.0221	397	438.2720	451	505.9898	505	572.3640	559	653.3023	613	703.3821
344	398.0263	398	439.1051	452	506.2902	506	573.0202	560	655.1099	614	704.1120
345	401.1419	399	439.2268	453	506.3294	507	573.2052	561	657.9775	615	705.1151
346	401.2476	400	442.0184	454	506.9932	508	576.0319	562	660.0135	616	707.3334
347	402.0122	401	443.0216	455	507.9686	509	577.3161	563	662.1586	617	708.1253
348	402.9963	402	445.0543	456	508.3058	510	578.3459	564	663.9855	618	709.0337
349	403.2633	403	445.9687	457	508.3421	511	579.0281	565	664.1188	619	710.0374
350	403.9838	404	447.1355	458	509.0277	512	579.2954	566	664.2917	620	711.4620
351	403.9997	405	447.2166	459	509.3091	513	580.9726	567	665.0700	621	713.3536
352	404.0161	406	447.3029	460	509.9612	514	581.3110	568	665.1051	622	714.3563
353	405.0748	407	448.0054	461	513.9756	515	582.3142	569	665.1226	623	715.5768
354	406.0115	408	448.9895	462	516.0196	516	585.0767	570	665.4047	624	719.4669
355	406.0203	409	449.0109	463	519.1505	517	586.0803	571	666.1237	625	720.3546
356	408.0129	410	449.0321	464	520.0267	518	587.0308	572	668.1138	626	721.3124
357	409.0162	411	449.3185	465	521.9847	519	590.3746	573	668.3254	627	721.4828
358	409.2374	412	450.3218	466	522.0447	520	591.2120	574	669.3289	628	722.5142
359	410.0169	413	452.0199	467	522.2392	521	591.3775	575	671.0867	629	723.3745
360	411.0013	414	452.2796	468	522.3257	522	592.2142	576	671.3395	630	723.4984
361	412.9367	415	461.0895	469	524.0603	523	595.0021	577	672.1043	631	724.3879
362	414.1007	416	462.3003	470	524.2795	524	597.3061	578	673.1285	632	724.5018
363	415.0326	417	463.2268	471	524.3430	525	599.3215	579	677.0918	633	725.0076
364	415.2269	418	463.2843	472	525.3461	526	600.3247	580	678.0981	634	725.3911
365	416.2302	419	463.9793	473	526.3453	527	600.3953	581	679.1019	635	725.5146
366	417.2424	420	464.0003	474	527.9718	528	601.3265	582	680.1138	636	727.0442
367	418.0019	421	466.0140	475	528.2744	529	603.3317	583	681.3997	637	727.3985
368	419.2581	422	466.0566	476	531.9432	530	604.0712	584	682.1294	638	728.4026
369	420.2615	423	466.9904	477	534.3208	531	606.0756	585	683.1332	639	734.1395
370	421.9941	424	468.0340	478	535.0065	532	606.3423	586	684.1380	640	735.3360
371	424.0079	425	470.0265	479	535.1244	533	606.3750	587	686.1013	641	735.3645
372	424.9784	426	473.3186	480	540.0552	534	607.0792	588	687.0518	642	737.3899
373	426.0131	427	474.0018	481	540.3378	535	608.2654	589	687.1072	643	738.5088
374	426.0235	428	474.3220	482	541.0587	536	610.9761	590	687.5456	644	739.3694
375	426.0351	429	475.3342	483	541.3412	537	611.0924	591	690.1149	645	741.3855
376	427.0094	430	477.3498	484	542.0603	538	611.1461	592	690.3072	646	741.9986
377	427.0309	431	478.2953	485	543.9457	539	612.1498	593	693.3174	647	742.5406
378	427.0571	432	478.3532	486	544.3327	540	616.3907	594	694.3408	648	744.0851

Supplementary Table S2. ***Continued from previous page***

#	m/z	#	m/z	#	m/z
649	745.3111	703	826.5759	757	933.5365
650	745.5036	704	827.3477	758	933.6022
651	746.3696	705	828.6041	759	934.5396
652	747.4985	706	829.6076	760	934.6053
653	747.5193	707	831.4594	761	937.5369
654	748.5019	708	832.4630	762	945.4885
655	748.5265	709	833.0971	763	949.5312
656	749.3900	710	833.5197	764	950.5345
657	749.5149	711	834.5311	765	991.2110
658	750.5184	712	835.5354	766	1032.5477
659	750.5457	713	842.3182	767	1071.1773
660	751.4932	714	851.1080	768	1093.1593
661	751.5301	715	852.1114	769	1167.7254
662	752.4968	716	853.4416	770	1168.7287
663	753.3850	717	854.4451	771	1185.7360
664	753.4218	718	855.4601	772	1186.7393
665	755.4298	719	856.0301	773	1189.7073
666	758.0644	720	856.4624	774	1207.7181
667	760.4687	721	856.6356	775	1208.7214
668	761.4036	722	857.5197	776	1209.7293
669	762.5091	723	857.6391	777	1210.7361
670	763.3949	724	858.5235	778	1223.6910
671	763.5124	725	859.5361	779	1447.9636
672	764.5254	726	860.5392	780	1448.9672
673	766.0672	727	861.5512	781	1449.9730
674	766.5405	728	862.5545	782	1450.9794
675	767.5439	729	863.5688	783	1451.9887
676	769.4163	730	864.5714	784	1469.9455
677	769.5037	731	867.0819	785	1470.9489
678	770.5717	732	869.4155	786	1471.9552
679	773.5348	733	871.4524	787	1472.9621
680	775.0540	734	872.4555	788	1473.9719
681	783.4613	735	881.5195	789	1474.9793
682	784.1511	736	882.4069	790	1485.9170
683	784.4648	737	883.5355	791	1486.9205
684	786.1671	738	884.5393	792	1487.9264
685	788.0489	739	885.5511	793	1495.9602
686	788.4998	740	886.5545	794	1497.9750
687	788.5249	741	887.5567		
688	788.5459	742	888.5581		
689	790.5405	743	894.5141		
690	791.4274	744	901.5465		
691	791.5439	745	905.5706		
692	792.5574	746	909.5510		
693	793.4162	747	909.6019		
694	794.3910	748	910.5544		
695	794.5356	749	911.5675		
696	794.5721	750	912.5697		
697	795.3951	751	913.5366		
698	795.5752	752	915.5257		
699	806.0583	753	917.5413		
700	810.5300	754	918.5445		
701	818.5355	755	927.4780		
702	826.3444	756	931.5203		

Supplementary Table S3. The top 10 discriminating features of each cluster in rat heart (listed in rows from left to right according to m/z size).

15 min	cluster 1	193.0781	194.0621	194.0820	195.0854	241.0629	255.0422	273.0350	385.1489	401.1419	431.1170
	cluster 2	187.0430	206.0867	208.0295	208.9860	262.1130	278.1079	306.0565	332.2277	348.0668	360.2590
	cluster 3	158.9265	238.8928	408.0129	482.9626	487.9793	505.9898	506.9932	521.9847	527.9718	543.9457
	cluster 4	328.0463	346.0585	347.0410	347.0601	402.9963	442.0184	448.0054	493.0538	540.0552	541.0587
	cluster 5	150.0241	206.0867	208.0295	278.1079	332.2277	360.2590	727.3985	760.4687	783.4613	784.4648
	cluster 6	124.0083	126.0041	181.9671	243.9673	271.0052	272.0084	273.0010	286.9791	599.3215	885.5511
30 min	cluster 1	193.0781	194.0621	194.0820	241.0629	255.0422	273.0350	370.1361	385.1489	401.1419	431.1170
	cluster 2	187.0430	206.0867	208.0295	208.9860	229.0549	262.1130	306.0565	332.2277	348.0668	360.2590
	cluster 3	158.9265	238.8928	408.0129	426.0235	429.9948	487.9793	505.9898	506.9932	527.9718	549.9537
	cluster 4	243.9579	328.0463	346.0585	347.0410	347.0601	360.9720	442.0184	448.0054	493.0538	595.0021
	cluster 5	206.0867	278.1079	306.0565	332.2277	348.0668	360.2590	668.3254	727.3985	755.4298	783.4613
	cluster 6	124.0083	243.9673	271.0052	272.0084	273.0010	286.9791	287.9824	299.0001	599.3215	682.1294
1 h	cluster 1	193.0781	194.0621	194.0820	195.0854	241.0629	255.0422	273.0350	370.1361	385.1489	401.1419
	cluster 2	187.0430	206.0139	208.0295	208.9860	262.1130	278.1079	306.0565	332.2277	348.0668	360.2590
	cluster 3	158.9265	191.0209	238.8928	408.0129	426.0235	487.9793	505.9898	506.9932	527.9718	543.9457
	cluster 4	211.0024	346.0585	347.0410	347.0601	362.0520	402.9963	448.0054	493.0538	540.0552	595.0021
	cluster 5	150.0241	206.0867	278.1079	668.3254	696.3567	724.3879	727.3985	755.4298	783.4613	784.4648
	cluster 6	124.0083	126.0041	243.9673	271.0052	272.0084	273.0010	286.9791	287.9824	599.3215	682.1294
2 h	cluster 1	193.0781	194.0621	194.0820	195.0854	241.0629	311.1711	325.1853	370.1361	385.1489	401.1419
	cluster 2	187.0430	206.0867	208.0295	208.9860	262.1130	278.1079	306.0565	332.2277	348.0668	360.2590
	cluster 3	158.9265	238.8928	408.0129	429.9948	487.9793	505.9898	506.9932	509.9612	527.9718	549.9537
	cluster 4	328.0463	346.0585	347.0601	402.9963	426.0235	442.0184	448.0054	493.0538	540.0552	565.0509
	cluster 5	150.0241	206.0867	508.3421	668.3254	696.3567	724.3879	727.3985	755.4298	783.4613	784.4648
	cluster 6	124.0083	271.0052	272.0084	273.0010	286.9791	419.2581	682.1294	861.5512	885.5511	886.5545
4 h	cluster 1	193.0781	194.0621	194.0820	195.0854	241.0629	255.0422	370.1361	385.1489	401.1419	431.1170
	cluster 2	187.0430	193.0781	194.0820	208.9860	229.0549	262.1130	278.1079	306.0565	332.2277	360.2590
	cluster 3	158.9265	238.8928	408.0129	426.0235	427.0309	487.9793	505.9898	506.9932	527.9718	549.9537
	cluster 4	328.0463	346.0585	347.0601	448.0054	493.0538	540.0552	541.0587	558.0658	595.0021	678.0981
	cluster 5	150.0241	206.0867	278.1079	524.3430	548.0410	696.3567	724.3879	727.3985	755.4298	783.4613
	cluster 6	271.0052	272.0084	273.0010	283.2654	419.2581	599.3215	600.3247	682.1294	861.5512	885.5511

Supplementary Table S4. Differentially expressed annotated and non-annotated features in response to ischemia (control ROI I vs. ischemic ROI I at each time point); statistical significance was determined by multivariate regression analysis using Benjamini-Hochberg's FDR<0.05; ND - no difference. NA – not applicable.

feature	pattern of expression	m/z	name	HMDB m/z	kegg	15min logFC	15min adj.P.Val	30 min logFC	30 min adj.P.Val	1 h logFC	1 h adj.P.Val	2 h logFC	2 h adj.P.Val	4 h logFC	4 h adj.P.Val
annotated	common to all	132.0312	L-aspartic acid	132.0302	C00049	-1.86	0.000	-1.51	0.000	-1.85	0.000	-2.47	0.000	-2.79	0.000
annotated	common to all	134.0482	Adenine	134.0472	C00147	-1.66	0.000	-1.85	0.000	-2.08	0.000	-1.91	0.000	-2.16	0.000
annotated	common to all	176.9370	Diphosphate (Pyrophosphate)	176.9360	C00013	-1.38	0.000	-1.00	0.002	-1.33	0.000	-1.12	0.001	-1.37	0.000
annotated	common to all	191.0209	Citric acid	191.0197	C00158	-3.32	0.000	-3.09	0.000	-3.54	0.000	-3.68	0.000	-3.65	0.000
annotated	common to all	236.0243	S-Cysteinosuccinic acid	236.0234		2.30	0.000	2.00	0.000	1.28	0.000	0.75	0.010	0.65	0.016
annotated	common to all	238.8928	Trimetaphosphoric acid	238.8917	C02466	-1.17	0.000	-1.05	0.000	-1.37	0.000	-1.36	0.000	-1.30	0.000
annotated	common to all	271.0148	1,3,5-trihydroxy-4-(sulfooxy)cyclohexane-1-carboxylic acid	271.0129		0.77	0.000	0.61	0.004	0.84	0.000	0.79	0.000	0.72	0.000
annotated	common to all	272.9582	Ribose-1-arsenate	272.9597		-1.36	0.003	-1.48	0.001	-1.77	0.000	-1.31	0.003	-1.83	0.000
annotated	common to all	273.0010	D-glucuronic acid 1-phosphate	273.0017	C05385	0.69	0.000	0.62	0.001	0.71	0.000	0.72	0.000	0.63	0.000
annotated	common to all	279.0279	1-Methoxyspirobrassinin	279.0267		-1.53	0.000	-1.32	0.000	-1.46	0.000	-1.84	0.000	-1.96	0.000
annotated	common to all	305.0245	2-(3,4-Dihydroxybenzoyloxy)-4,6-dihydroxybenzoic acid	305.0222	C04524	-0.90	0.002	-0.77	0.009	-1.08	0.000	-1.12	0.000	-1.17	0.000
annotated	common to all	315.0498	Isorhamnetin	315.0510	C10084	0.68	0.041	0.78	0.014	0.81	0.010	0.80	0.011	1.10	0.000
annotated	common to all	328.0463	Cyclic AMP	328.0452	C00575	-1.57	0.000	-1.76	0.000	-1.96	0.000	-1.48	0.001	-1.93	0.000
annotated	common to all	329.0508	6-(2-carboxy-5-hydroxyphenoxy)-3,4,5-trihydroxyoxane-2-carboxylic acid	329.0489		-1.49	0.000	-1.59	0.000	-1.84	0.000	-1.50	0.000	-1.87	0.000
annotated	common to all	338.9900	Fructose 1,6-bisphosphate/1D-Myo-inositol 1,3-bisphosphate/ Alpha-D-Glucose 1,6-bisphosphate	338.9888	C00354/ C01231	-3.27	0.000	-3.80	0.000	-4.07	0.000	-2.41	0.001	-3.62	0.000
annotated	common to all	344.0413	Cyclic GMP	344.0402	C00942	-1.90	0.000	-1.81	0.000	-2.06	0.000	-2.01	0.000	-2.03	0.000
annotated	common to all	346.0585	Adenosine monophosphate (AMP)	346.0558	C00020	-0.81	0.018	-1.08	0.002	-1.87	0.000	-1.94	0.000	-2.46	0.000
annotated	common to all	347.0410	Inosinic acid (IMP)	347.0398	C00130	-4.37	0.000	-3.40	0.000	-2.54	0.001	-1.97	0.009	-1.85	0.009
annotated	common to all	362.0520	Guanosine monophosphate (GMP)	362.0507	C00144	-1.39	0.002	-1.68	0.000	-1.74	0.000	-1.62	0.000	-1.96	0.000
annotated	common to all	402.0122	Cytidine diphosphate (CDP)	402.0109	C00112	-2.03	0.000	-1.89	0.000	-2.20	0.000	-1.98	0.000	-2.21	0.000
annotated	common to all	402.9963	Uridine 5'-diphosphate (UDP)	402.9949	C00015	-1.79	0.000	-1.61	0.000	-2.29	0.000	-2.44	0.000	-3.75	0.000
annotated	common to all	426.0131	Adenosine phosphosulfate	426.0126	C00224	-1.61	0.010	-1.86	0.003	-2.15	0.000	-2.13	0.001	-2.12	0.000
annotated	common to all	426.0235	Adp	426.0221	C00008	-2.51	0.000	-2.89	0.000	-2.97	0.000	-2.44	0.000	-2.99	0.000
annotated	common to all	427.0094	IDP	427.0062	C00104	-1.87	0.000	-1.75	0.000	-1.94	0.000	-1.76	0.000	-1.90	0.000
annotated	common to all	442.0184	Guanosine diphosphate/8-oxo-dGDP	442.0171	C00035/C 20176	-3.35	0.000	-3.83	0.000	-3.65	0.000	-2.99	0.000	-3.07	0.000
annotated	common to all	482.9626	Uridine triphosphate (UTP)	482.9613	C00075	-0.95	0.002	-0.82	0.008	-1.15	0.000	-1.16	0.000	-1.08	0.000
annotated	common to all	505.9898	Adenosine triphosphate (ATP)	505.9885	C00002	-3.35	0.000	-3.91	0.000	-3.76	0.000	-3.06	0.000	-3.33	0.000
annotated	common to all	521.9847	Guanosine triphosphate (GTP)	521.9834	C00044	-1.95	0.000	-1.86	0.000	-2.15	0.000	-2.07	0.000	-2.06	0.000
annotated	common to all	565.0509	Uridine diphosphate glucose	565.0477	C00029	-2.40	0.000	-2.59	0.000	-3.76	0.000	-3.68	0.000	-4.60	0.000
annotated	common to all	573.0202	Gossypetin 8-glucuronide 3-sulfate	573.0192		-2.29	0.000	-2.56	0.000	-2.71	0.000	-2.44	0.000	-2.72	0.000
annotated	common to all	620.0215	ADP-ribose 1''-2'' cyclic phosphate	620.0201		-1.35	0.006	-1.61	0.001	-1.98	0.000	-1.58	0.001	-2.19	0.000
annotated	common to all	638.0321	ADP-ribose 2'-phosphate	638.0307	C03246	-0.80	0.039	-0.97	0.009	-1.22	0.001	-1.30	0.001	-1.38	0.000
annotated	common to all	682.1294	(S)-NADHX	682.1281	C04856	2.79	0.000	3.97	0.000	4.98	0.000	5.65	0.000	6.15	0.000
annotated	common to all	271.0245	[2-methoxy-4-(3-oxobut-1-en-1-yl)phenyl]oxidanesulfonic acid	271.0282	C05385	0.62	0.003	0.51	0.014	0.71	0.000	0.68	0.001	0.57	0.003
annotated	common to all	272.0084	Stepronin	272.0057		0.72	0.000	0.62	0.001	0.73	0.000	0.74	0.000	0.64	0.000
annotated	common to all	293.0436	Wasalexin A	293.0424		1.15	0.000	1.15	0.000	1.06	0.000	0.90	0.000	0.93	0.000
annotated	common to all	300.0898	Citrusinine I	300.0877		0.98	0.041	0.91	0.049	0.93	0.045	0.93	0.041	1.13	0.008

feature	pattern of expression	m/z	name	HMDB m/z	kegg	15min logFC	15min adj.P.Val	30 min logFC	30 min adj.P.Val	1 h logFC	1 h adj.P.Val	2 h logFC	2 h adj.P.Val	4 h logFC	4 h adj.P.Val
annotated	common to all	427.0309	{4-[2,3-dioxo-3-(2,4,6-trihydroxy-3-methoxyphenyl)propyl]-2-methoxyphenyl}oxidanesulfonic acid	427.0341		-2.56	0.000	-2.90	0.000	-3.00	0.000	-2.49	0.000	-3.03	0.000
annotated	common to all	428.0276	Cloransulam-methyl	428.0237		-2.22	0.000	-2.31	0.000	-2.54	0.000	-2.22	0.000	-2.62	0.000
annotated	other (15m,1h,2h,4h)	322.0505	Cytidine monophosphate (18ppm)	322.0446	C00055	-0.66	0.013	ND	ND	-0.68	0.009	-0.59	0.024	-0.69	0.005
annotated	other (15m,30m,1h)	146.0469	L-Glutamic acid (Glutamate)	146.0459	C00025	1.01	0.002	1.14	0.000	0.76	0.017	ND	ND	ND	ND
annotated	other (15m,30m,1h)	352.9509	Pipobroman	352.9506	C07362	1.07	0.001	0.87	0.009	0.90	0.006	ND	ND	ND	ND
annotated	other (15m,30m,2h,4h)	171.0074	Glycerol 3-phosphate	171.0064	C00093	0.77	0.019	1.02	0.002	ND	ND	0.86	0.006	0.70	0.018
annotated	other (15m,30m)	124.0083	Taurine	124.0074	C00245	0.40	0.043	0.47	0.013	ND	ND	ND	ND	ND	ND
annotated	other (15m,30m)	167.9983	Cysteic acid	167.9972	C00506	0.50	0.039	0.55	0.016	ND	ND	ND	ND	ND	ND
annotated	other (15m,30m)	125.0077	2-Acetylthiophene	125.0067		0.43	0.034	0.52	0.008	ND	ND	ND	ND	ND	ND
annotated	other (15m,4h)	677.0918	5-Phosphoribosyl-4-carboxy-5-aminoimidazole	677.0917		0.73	0.041	ND	ND	ND	ND	ND	ND	0.90	0.006
annotated	other (1h,2h,4h)	138.9812	Phosphonoacetic acid	138.9802	C05682	ND	ND	ND	ND	-0.82	0.008	-0.62	0.047	-0.61	0.034
annotated	other (1h,2h,4h)	297.0392	Irilone	297.0405		ND	ND	ND	ND	0.54	0.037	0.63	0.012	0.74	0.002
annotated	other (1h,2h,4h)	335.0663	S-Nitrosoglutathione	335.0667		ND	ND	ND	ND	0.63	0.047	1.43	0.000	2.10	0.000
annotated	other (1h,2h,4h)	558.0658	Adenosine diphosphate ribose	558.0644	C00301	ND	ND	ND	ND	-1.14	0.012	-1.03	0.024	-1.60	0.000
annotated	other (1h,2h,4h)	597.3061	1-Oleoylglycerophosphoinositol	597.3045		ND	ND	ND	ND	0.75	0.010	0.70	0.017	1.31	0.000
annotated	other (1h,2h,4h)	859.5361	Pi(16:0/20:3(8Z,11Z,14Z))	859.5342	C00626	ND	ND	ND	ND	1.12	0.035	1.18	0.024	1.40	0.005
annotated	other (1h,2h,4h)	415.0326	5-{[(2E)-3-(3,4-dihydroxyphenyl)prop-2-enoyl]oxy}-4-hydroxy-3-(sulfooxy)cyclohex-1-ene-1-carboxylic acid	415.0341		ND	ND	ND	ND	1.57	0.000	2.26	0.000	2.77	0.000
annotated	other (1h,2h,4h)	474.0018	Dideoxyadenosine Triphosphate	473.9986		ND	ND	ND	ND	-0.97	0.015	-1.03	0.009	-1.03	0.006
annotated	other (1h,2h,4h)	411.0013	[2-hydroxy-5-(3,5,7-trihydroxy-6-methoxy-4-oxo-4H-chromen-2-yl)phenyl]oxidanesulfonic acid	411.0028		ND	ND	ND	ND	-1.30	0.013	-1.18	0.024	-1.73	0.001
annotated	other (1h,4h)	151.0273	Xanthine	151.0261	C00385	ND	ND	ND	ND	1.35	0.006	ND	ND	1.27	0.006
annotated	other (1h,4h)	678.0981	UDP-N-acetylmuraminate	678.0954	C01050	ND	ND	ND	ND	-1.67	0.034	ND	ND	-2.04	0.006
annotated	other (2h,4h)	211.1350	Traumatin	211.1340	C16309	ND	ND	ND	ND	ND	ND	0.75	0.015	0.94	0.002
annotated	other (2h,4h)	414.1007	Cephalosporin C	414.0977	C00916	ND	ND	ND	ND	ND	ND	0.77	0.023	0.70	0.027
annotated	other (2h,4h)	417.2424	CPA(18:1(11Z)/0:0)	417.2411		ND	ND	ND	ND	ND	ND	0.61	0.035	0.73	0.007
annotated	other (2h,4h)	419.2581	CPA(18:0/0:0)	419.2568		ND	ND	ND	ND	ND	ND	0.47	0.035	0.59	0.005
annotated	other (2h,4h)	606.0756	Uridine diphosphate-N-acetylglucosamine	606.0743	C00043	ND	ND	ND	ND	ND	ND	-1.21	0.006	-2.94	0.000
annotated	other (2h,4h)	664.1188	NADH	664.1175	C00004	ND	ND	ND	ND	ND	ND	1.62	0.001	1.96	0.000
annotated	other (2h,4h)	664.2917	Natamycin	664.2909	C08073	ND	ND	ND	ND	ND	ND	0.56	0.041	0.54	0.033
annotated	other (2h,4h)	744.0851	NADPH	744.0838	C00005	ND	ND	ND	ND	ND	ND	1.20	0.002	1.53	0.000
annotated	other (2h,4h)	852.1114	Malonyl-CoA	852.1083	C00083	ND	ND	ND	ND	ND	ND	0.62	0.035	1.25	0.000
annotated	other (2h,4h)	861.5512	Pi(20:2(11Z,14Z)/16:0)	861.5499		ND	ND	ND	ND	ND	ND	1.03	0.036	1.24	0.007
annotated	other (2h,4h)	862.5545	PS(20:2(11Z,14Z)/22:4(7Z,10Z,13Z,16Z))	862.5604		ND	ND	ND	ND	ND	ND	1.08	0.034	1.29	0.007
annotated	other (2h,4h)	397.0221	[4-(5,7-dihydroxy-4-oxo-3,4-dihydro-2H-1-benzopyran-2-yl)-2-hydroxy-6-methoxyphenyl]oxidanesulfonic acid	397.0235		ND	ND	ND	ND	ND	ND	1.40	0.002	1.76	0.000
annotated	other (2h,4h)	707.3334	Scillipheosidin 3-[glucosyl-(1->2)-rhamnoside]	707.3284		ND	ND	ND	ND	ND	ND	0.87	0.008	1.12	0.001
annotated	other (2h,4h)	755.4298	Tuberoside J	755.4223		ND	ND	ND	ND	ND	ND	0.88	0.015	0.78	0.021
annotated	other (2h,4h)	783.4613	Hoduloside VI	783.4536		ND	ND	ND	ND	ND	ND	0.94	0.045	0.93	0.033
annotated	other (30m,1h,2h,4h)	230.0116	Benzeneacetamide-4-O-sulphate	230.0129		ND	ND	0.82	0.007	0.62	0.041	0.59	0.050	0.62	0.026
annotated	other (30m,1h,2h,4h)	346.0473	5'-Hydroxypiroxicam	346.0463		ND	ND	-0.58	0.032	-1.00	0.000	-0.92	0.001	-0.99	0.000
annotated	other (30m,1h,2h,4h)	599.3215	LysoPI(18:0/0:0)	599.3202		ND	ND	1.05	0.011	1.12	0.006	1.09	0.007	1.59	0.000
annotated	other (30m,1h,2h,4h)	627.1235	(3-{3,5,7-trihydroxy-8-[2-hydroxy-3-(3-hydroxyphenyl)-1-(2,4,6-trihydroxyphenyl)propyl]-3,4-dihydro-2H-1-benzopyran-2-yl}phenyl)oxidanesulfonic acid	627.1178		ND	ND	1.13	0.023	1.96	0.000	3.45	0.000	2.87	0.000

feature	pattern of expression	m/z	name	HMDB m/z	kegg	15min logFC	15min adj.P.Val	30 min logFC	30 min adj.P.Val	1 h logFC	1 h adj.P.Val	2 h logFC	2 h adj.P.Val	4 h logFC	4 h adj.P.Val
annotated	other (30m,1h,2h)	786.1671	FADH	786.1655	C01352	ND	ND	-0.87	0.029	-1.02	0.010	-0.81	0.041	ND	ND
annotated	other (30m,1h,4h)	571.2903	1-Palmitoylglycerophosphoinositol	571.2889		ND	ND	0.89	0.022	0.86	0.027	ND	ND	1.24	0.001
annotated	other (30m,1h)	784.1511	FAD	784.1499	C00016	ND	ND	-0.83	0.048	-0.96	0.019	ND	ND	ND	ND
annotated	other (30m,2h)	445.0543	CDP-Ethanolamine	445.0531	C00570	ND	ND	1.63	0.011	ND	ND	1.33	0.035	ND	ND
annotated	other (30m,4h)	463.2843	1-(11Z-eicosenoyl)-glycero-3-phosphate	463.2830		ND	ND	0.69	0.037	ND	ND	ND	ND	0.77	0.012
annotated	unique to 2h	640.2941	S-(9-deoxy-delta9,12-PGD2)-glutathione	640.2909		ND	ND	ND	ND	ND	ND	0.69	0.034	ND	ND
annotated	unique to 30 min	324.9794	Niclosamide	324.9788		ND	ND	0.93	0.015	ND	ND	ND	ND	ND	ND
annotated	unique to 30 min	365.0922	Atovaquone	365.0950	C06835	ND	ND	2.01	0.009	ND	ND	ND	ND	ND	ND
annotated	unique to 30 min	316.0638	5,7,18,20-Tetraoxa-24-azahexacyclo[11.11.0.0Â²,Â¹â°.0â´,â¸.0Â¹â´,Â²Â².0Â¹â·,Â²Â¹]tetracosa-1(24),2,4(8),9,11,13,15,17(21),22-nonaene	316.0615	C05191	ND	ND	1.18	0.014	ND	ND	ND	ND	ND	ND
annotated	unique to 30 min	207.0452	Cysteinyl-Serine	207.0445		ND	ND	1.33	0.012	ND	ND	ND	ND	ND	ND
annotated	unique to 4h	140.0129	O-Phosphoethanolamine	140.0118	C00346	ND	ND	ND	ND	ND	ND	ND	ND	0.58	0.016
annotated	unique to 4h	171.1037	9-Oxononanoic acid	171.1027	C16322	ND	ND	ND	ND	ND	ND	ND	ND	0.86	0.022
annotated	unique to 4h	224.0705	Murrayanine	224.0717	C09228	ND	ND	ND	ND	ND	ND	ND	ND	0.92	0.005
annotated	unique to 4h	241.0131	Inositol cyclic phosphate	241.0119		ND	ND	ND	ND	ND	ND	ND	ND	0.45	0.024
annotated	unique to 4h	255.2341	Palmitic acid	255.2330	C00249	ND	ND	ND	ND	ND	ND	ND	ND	0.55	0.014
annotated	unique to 4h	269.2498	Methyl hexadecanoic acid	269.2486	C16995	ND	ND	ND	ND	ND	ND	ND	ND	0.61	0.006
annotated	unique to 4h	281.2497	Oleic acid	281.2486	C00712	ND	ND	ND	ND	ND	ND	ND	ND	0.75	0.024
annotated	unique to 4h	283.2654	Stearic acid	283.2643	C01530	ND	ND	ND	ND	ND	ND	ND	ND	0.58	0.010
annotated	unique to 4h	317.0572	Melanin	317.0568	C05606	ND	ND	ND	ND	ND	ND	ND	ND	0.76	0.005
annotated	unique to 4h	391.2268	CPA(16:0/0:0)	391.2255		ND	ND	ND	ND	ND	ND	ND	ND	0.58	0.009
annotated	unique to 4h	435.2530	DHAP(18:0)/LPA(18:1(9Z)/0:0)	435.2517	C03805/C00416	ND	ND	ND	ND	ND	ND	ND	ND	0.53	0.036
annotated	unique to 4h	478.2953	Lysope(0:0/18:1(9Z))	478.2939		ND	ND	ND	ND	ND	ND	ND	ND	0.59	0.027
annotated	unique to 4h	480.3108	Lysopc(15:0)	480.3096	C04230	ND	ND	ND	ND	ND	ND	ND	ND	0.68	0.010
annotated	unique to 4h	508.3421	PC(17:0/0:0)	508.3409	C04230	ND	ND	ND	ND	ND	ND	ND	ND	0.76	0.022
annotated	unique to 4h	579.0281	Uridine diphosphate glucuronic acid	579.0270	C00167	ND	ND	ND	ND	ND	ND	ND	ND	-1.04	0.011
annotated	unique to 4h	655.1099	Gossypetin 8-glucuronide 3-glucoside	655.1060		ND	ND	ND	ND	ND	ND	ND	ND	0.91	0.012
annotated	unique to 4h	699.4986	PA(18:0/18:2(9Z,12Z))	699.4970	C00416	ND	ND	ND	ND	ND	ND	ND	ND	0.78	0.040
annotated	unique to 4h	747.5193	PG(16:0/18:1(11Z))	747.5182		ND	ND	ND	ND	ND	ND	ND	ND	0.74	0.042
annotated	unique to 4h	748.5265	PE(P-18:1(9Z)/20:4(5Z,8Z,11Z,14Z))	748.5287	C00350	ND	ND	ND	ND	ND	ND	ND	ND	0.72	0.036
annotated	unique to 4h	769.5037	PG(16:0/20:4(5Z,8Z,11Z,14Z))	769.5025		ND	ND	ND	ND	ND	ND	ND	ND	0.80	0.047
annotated	unique to 4h	833.5197	Pi(18:1(9Z)/16:1(9Z))	833.5186	C00626	ND	ND	ND	ND	ND	ND	ND	ND	1.14	0.017
annotated	unique to 4h	857.5197	Pi(20:4(5Z,8Z,11Z,14Z)/16:0)	857.5186		ND	ND	ND	ND	ND	ND	ND	ND	1.28	0.047
annotated	unique to 4h	871.4524	PGP(18:3(6Z,9Z,12Z)/20:4(5Z,8Z,11Z,14Z))	871.4532		ND	ND	ND	ND	ND	ND	ND	ND	0.96	0.017
annotated	unique to 4h	883.5355	Pi(20:4(5Z,8Z,11Z,14Z)/18:1(9Z))	883.5342		ND	ND	ND	ND	ND	ND	ND	ND	1.40	0.039
annotated	unique to 4h	209.0806	2,2-dimethyl-3,4-dihydro-2H-1-benzopyran-4,5,7-triol	209.0819		ND	ND	ND	ND	ND	ND	ND	ND	1.07	0.041
annotated	unique to 4h	231.0438	3-Chloro-1-(4-hydroxy-3-methoxyphenyl)propane-1,2-diol	231.0430		ND	ND	ND	ND	ND	ND	ND	ND	1.26	0.000
annotated	unique to 4h	239.0355	Xanthopurpurin	239.0350	C10370	ND	ND	ND	ND	ND	ND	ND	ND	-0.53	0.036
annotated	unique to 4h	277.2185	Gamma-Linolenic acid	277.2173	C06426	ND	ND	ND	ND	ND	ND	ND	ND	0.58	0.022
annotated	unique to 4h	298.0710	D-4'-Phosphopantothenate	298.0697	C03492	ND	ND	ND	ND	ND	ND	ND	ND	1.39	0.000
annotated	unique to 4h	314.0190	Efavirenz	314.0201	C08088	ND	ND	ND	ND	ND	ND	ND	ND	0.53	0.033
annotated	unique to 4h	320.0529	Clopidogrel	320.0518		ND	ND	ND	ND	ND	ND	ND	ND	1.02	0.000
annotated	unique to 4h	325.1092	7-hydroxy-1,7-bis(4-hydroxyphenyl)hept-1-ene-3,5-dione	325.1081		ND	ND	ND	ND	ND	ND	ND	ND	1.85	0.002
annotated	unique to 4h	328.1211	N-trans-Feruloyloctopamine	328.1190		ND	ND	ND	ND	ND	ND	ND	ND	1.13	0.006

feature	pattern of expression	m/z	name	HMDB m/z	kegg	15min logFC	15min adj.P.Val	30 min logFC	30 min adj.P.Val	1 h logFC	1 h adj.P.Val	2 h logFC	2 h adj.P.Val	4 h logFC	4 h adj.P.Val
annotated	unique to 4h	353.0819	Niazidin	353.0813		ND	ND	ND	ND	ND	ND	ND	ND	1.02	0.000
annotated	unique to 4h	452.2796	Lysope(16:0/0:0)	452.2783		ND	ND	ND	ND	ND	ND	ND	ND	0.66	0.015
annotated	unique to 4h	573.2052	Tetrahydrofolyl-[Glu](2)	573.2063		ND	ND	ND	ND	ND	ND	ND	ND	0.88	0.004
annotated	unique to 4h	591.2120	8-Acetoxy-4'-methoxypinoresinol 4-glucoside	591.2083		ND	ND	ND	ND	ND	ND	ND	ND	1.39	0.006
annotated	unique to 4h	619.2902	1-Arachidonoylglycerophosphoinositol	619.2889		ND	ND	ND	ND	ND	ND	ND	ND	0.68	0.014
annotated	unique to 4h	687.1072	4-[11-hydroxy-7-(4-hydroxy-3,5-dimethoxyphenyl)-6-{[3,4,5-trihydroxy-6-(hydroxymethyl)oxan-2-yl]oxy}-2,8-dioxatricyclo[7.3.1.0^{5},^{13}]trideca-1(13),4,6,9,11-pentaen-3-ylidene]cyclohexa-2,5-dien-1-ylidene hydrogen sulfuric acidium	687.1025		ND	ND	ND	ND	ND	ND	ND	ND	0.60	0.041
annotated	unique to 4h	763.3949	Spinacoside D	763.3910		ND	ND	ND	ND	ND	ND	ND	ND	0.66	0.007
annotated	unique to 4h	791.4274	Mabioside C	791.4223		ND	ND	ND	ND	ND	ND	ND	ND	0.91	0.001
annotated	unique to 4h	860.5392	PS(22:4(7Z,10Z,13Z,16Z)/20:3(5Z,8Z,11Z))	860.5447		ND	ND	ND	ND	ND	ND	ND	ND	1.07	0.017
non-annotated	common to all	150.9813		NA		-0.95	0.002	-0.97	0.001	-1.16	0.000	-0.90	0.002	-0.96	0.001
non-annotated	common to all	158.9265		NA		-2.15	0.000	-2.18	0.000	-2.34	0.000	-1.92	0.000	-2.19	0.000
non-annotated	common to all	174.9813		NA		-0.78	0.001	-0.79	0.001	-0.97	0.000	-0.86	0.000	-0.94	0.000
non-annotated	common to all	180.9084		NA		-1.06	0.000	-0.99	0.000	-1.22	0.000	-1.18	0.000	-1.01	0.000
non-annotated	common to all	192.9948		NA		-1.30	0.000	-1.35	0.000	-1.61	0.000	-1.42	0.000	-1.71	0.000
non-annotated	common to all	198.9094		NA		-1.01	0.000	-0.92	0.001	-1.14	0.000	-1.10	0.000	-0.86	0.001
non-annotated	common to all	198.9190		NA		-1.70	0.000	-1.87	0.000	-1.94	0.000	-1.49	0.000	-1.70	0.000
non-annotated	common to all	211.0024		NA		-1.48	0.000	-1.51	0.000	-1.73	0.000	-1.62	0.000	-1.86	0.000
non-annotated	common to all	225.9568		NA		-0.81	0.001	-0.88	0.000	-0.83	0.000	-0.63	0.008	-0.61	0.007
non-annotated	common to all	271.0052		NA		0.67	0.000	0.60	0.001	0.67	0.000	0.68	0.000	0.60	0.001
non-annotated	common to all	273.0122		NA		0.71	0.000	0.61	0.001	0.73	0.000	0.75	0.000	0.65	0.000
non-annotated	common to all	274.0072		NA		0.69	0.000	0.67	0.000	0.72	0.000	0.73	0.000	0.63	0.000
non-annotated	common to all	274.9968		NA		0.47	0.013	0.45	0.015	0.48	0.008	0.51	0.005	0.41	0.017
non-annotated	common to all	290.9688		NA		-1.38	0.000	-1.24	0.000	-1.27	0.000	-1.50	0.000	-2.22	0.000
non-annotated	common to all	294.9402		NA		-1.27	0.001	-1.41	0.000	-1.40	0.000	-1.29	0.000	-1.27	0.000
non-annotated	common to all	312.9508		NA		-1.63	0.001	-1.96	0.000	-1.78	0.000	-1.47	0.002	-1.52	0.001
non-annotated	common to all	320.9794		NA		-1.71	0.000	-1.68	0.000	-2.06	0.000	-1.96	0.000	-2.12	0.000
non-annotated	common to all	324.0332		NA		-1.47	0.000	-1.41	0.000	-1.74	0.000	-1.78	0.000	-1.75	0.000
non-annotated	common to all	342.9614		NA		-1.67	0.000	-1.84	0.000	-2.21	0.000	-1.96	0.000	-2.30	0.000
non-annotated	common to all	347.0601		NA		-0.82	0.019	-1.11	0.001	-1.88	0.000	-1.98	0.000	-2.51	0.000
non-annotated	common to all	348.0444		NA		-3.29	0.000	-2.94	0.000	-2.44	0.000	-1.98	0.001	-1.76	0.003
non-annotated	common to all	349.0453		NA		-1.25	0.001	-1.25	0.001	-1.40	0.000	-1.13	0.002	-1.51	0.000
non-annotated	common to all	360.9720		NA		-3.11	0.000	-4.03	0.000	-3.90	0.000	-2.18	0.005	-3.22	0.000
non-annotated	common to all	361.9753		NA		-0.97	0.006	-0.91	0.010	-1.22	0.000	-1.17	0.001	-1.16	0.001
non-annotated	common to all	376.9460		NA		-2.41	0.000	-2.82	0.000	-3.06	0.000	-2.18	0.000	-3.12	0.000
non-annotated	common to all	384.0340		NA		-0.96	0.001	-0.84	0.002	-1.07	0.000	-1.20	0.000	-1.00	0.000
non-annotated	common to all	384.9857		NA		-1.15	0.010	-0.96	0.031	-1.67	0.000	-2.01	0.000	-3.46	0.000
non-annotated	common to all	384.9969		NA		-0.91	0.002	-0.79	0.006	-0.96	0.001	-1.00	0.001	-0.94	0.001
non-annotated	common to all	403.9997		NA		-1.63	0.000	-1.47	0.000	-1.95	0.000	-2.04	0.000	-2.42	0.000
non-annotated	common to all	404.0161		NA		-0.94	0.016	-1.51	0.000	-2.04	0.000	-2.17	0.000	-2.16	0.000
non-annotated	common to all	408.0129		NA		-1.38	0.002	-1.52	0.001	-1.63	0.000	-1.11	0.008	-1.39	0.001
non-annotated	common to all	409.0162		NA		-1.34	0.001	-1.43	0.001	-1.51	0.000	-1.08	0.007	-1.36	0.001
non-annotated	common to all	410.0169		NA		-0.77	0.002	-0.72	0.003	-0.96	0.000	-0.82	0.001	-0.96	0.000
non-annotated	common to all	418.0019		NA		1.05	0.000	0.71	0.003	0.92	0.000	0.93	0.000	0.94	0.000
non-annotated	common to all	424.0079		NA		-2.43	0.000	-2.43	0.000	-2.56	0.000	-2.29	0.000	-2.48	0.000

feature	pattern of expression	m/z	name	HMDB m/z	kegg	15min logFC	15min adj.P.Val	30 min logFC	30 min adj.P.Val	1 h logFC	1 h adj.P.Val	2 h logFC	2 h adj.P.Val	4 h logFC	4 h adj.P.Val
non-annotated	common to all	424.9784		NA		-1.44	0.001	-1.48	0.000	-1.81	0.000	-1.98	0.000	-2.43	0.000
non-annotated	common to all	426.0351		NA		-1.25	0.000	-1.18	0.000	-1.50	0.000	-1.43	0.000	-1.44	0.000
non-annotated	common to all	429.9948		NA		-1.62	0.002	-2.08	0.000	-1.91	0.000	-1.54	0.002	-1.56	0.002
non-annotated	common to all	430.9980		NA		-1.21	0.000	-1.24	0.000	-1.35	0.000	-1.30	0.000	-1.31	0.000
non-annotated	common to all	443.0216		NA		-2.02	0.000	-1.94	0.000	-2.15	0.000	-2.09	0.000	-2.09	0.000
non-annotated	common to all	445.9687		NA		-1.26	0.000	-1.29	0.000	-1.52	0.000	-1.48	0.000	-1.85	0.000
non-annotated	common to all	448.0054		NA		-1.47	0.001	-1.87	0.000	-1.64	0.000	-1.30	0.003	-1.37	0.001
non-annotated	common to all	448.9895		NA		-1.45	0.000	-1.32	0.000	-1.56	0.000	-1.61	0.000	-1.45	0.000
non-annotated	common to all	449.0109		NA		-1.49	0.001	-1.86	0.000	-1.63	0.000	-1.31	0.003	-1.39	0.001
non-annotated	common to all	452.0199		NA		-1.58	0.009	-1.65	0.006	-2.12	0.000	-2.06	0.001	-2.21	0.000
non-annotated	common to all	463.9793		NA		-1.19	0.000	-1.26	0.000	-1.31	0.000	-1.35	0.000	-2.13	0.000
non-annotated	common to all	464.0003		NA		-2.28	0.000	-2.28	0.000	-2.43	0.000	-2.34	0.000	-2.22	0.000
non-annotated	common to all	466.0140		NA		-1.22	0.000	-1.61	0.000	-2.12	0.000	-2.15	0.000	-2.23	0.000
non-annotated	common to all	466.9904		NA		-1.21	0.001	-1.08	0.002	-1.24	0.000	-1.18	0.001	-0.92	0.004
non-annotated	common to all	470.0265		NA		-1.32	0.000	-1.32	0.000	-1.61	0.000	-1.65	0.000	-1.60	0.000
non-annotated	common to all	487.9793		NA		-1.92	0.000	-2.05	0.000	-2.27	0.000	-2.03	0.000	-2.27	0.000
non-annotated	common to all	494.0379		NA		-3.57	0.000	-3.14	0.000	-2.34	0.001	-1.71	0.014	-1.41	0.030
non-annotated	common to all	506.9932		NA		-2.05	0.000	-2.03	0.000	-2.32	0.000	-2.20	0.000	-2.22	0.000
non-annotated	common to all	507.9686		NA		-1.53	0.000	-1.74	0.000	-2.00	0.000	-1.46	0.000	-1.72	0.000
non-annotated	common to all	509.9612		NA		-1.63	0.000	-1.84	0.000	-1.88	0.000	-1.66	0.000	-1.71	0.000
non-annotated	common to all	513.9756		NA		-1.00	0.007	-0.92	0.013	-1.25	0.001	-1.26	0.001	-1.18	0.001
non-annotated	common to all	522.0447		NA		-0.78	0.010	-0.75	0.014	-1.19	0.000	-0.93	0.002	-1.34	0.000
non-annotated	common to all	527.9718		NA		-2.95	0.000	-3.21	0.000	-3.26	0.000	-2.83	0.000	-2.94	0.000
non-annotated	common to all	531.9432		NA		-1.05	0.000	-0.99	0.000	-1.16	0.000	-1.20	0.000	-1.16	0.000
non-annotated	common to all	543.9457		NA		-1.72	0.000	-1.69	0.000	-2.04	0.000	-2.01	0.000	-2.01	0.000
non-annotated	common to all	545.9729		NA		-1.41	0.000	-1.32	0.000	-1.63	0.000	-1.62	0.000	-1.57	0.000
non-annotated	common to all	549.9537		NA		-2.11	0.000	-2.59	0.000	-2.44	0.000	-2.10	0.000	-2.05	0.000
non-annotated	common to all	564.9986		NA		1.22	0.000	0.73	0.005	0.98	0.000	1.03	0.000	1.10	0.000
non-annotated	common to all	565.9277		NA		-1.82	0.000	-1.88	0.000	-2.07	0.000	-2.07	0.000	-2.33	0.000
non-annotated	common to all	567.9553		NA		-1.61	0.000	-1.54	0.000	-1.74	0.000	-1.67	0.000	-1.66	0.000
non-annotated	common to all	587.0308		NA		-1.34	0.000	-1.24	0.000	-1.59	0.000	-1.61	0.000	-1.54	0.000
non-annotated	common to all	595.0021		NA		-1.82	0.001	-2.54	0.000	-2.28	0.000	-1.76	0.001	-2.03	0.000
non-annotated	common to all	610.9761		NA		-1.67	0.000	-1.90	0.000	-2.09	0.000	-1.96	0.000	-2.44	0.000
non-annotated	common to all	642.0035		NA		-1.40	0.004	-1.75	0.000	-1.91	0.000	-1.41	0.003	-1.89	0.000
non-annotated	common to all	651.9021		NA		-1.10	0.000	-0.99	0.000	-1.30	0.000	-1.31	0.000	-1.24	0.000
non-annotated	common to all	657.9775		NA		-1.06	0.001	-0.99	0.002	-1.46	0.000	-1.33	0.000	-1.79	0.000
non-annotated	common to all	660.0135		NA		-0.72	0.042	-0.76	0.028	-1.00	0.003	-0.93	0.006	-1.18	0.000
non-annotated	common to all	662.1586		NA		-1.06	0.000	-0.93	0.001	-0.99	0.000	-0.81	0.003	-1.00	0.000
non-annotated	common to all	663.9855		NA		-1.09	0.004	-1.20	0.002	-1.37	0.000	-1.17	0.002	-1.36	0.000
non-annotated	common to all	683.1332		NA		1.05	0.006	2.24	0.000	3.25	0.000	3.91	0.000	4.41	0.000
non-annotated	common to all	696.9504		NA		-0.77	0.001	-0.67	0.004	-0.98	0.000	-0.97	0.000	-0.91	0.000
non-annotated	common to all	702.0979		NA		-0.84	0.002	-0.80	0.003	-1.02	0.000	-0.97	0.000	-1.08	0.000
non-annotated	common to all	725.3911		NA		0.68	0.017	0.60	0.031	0.62	0.027	0.83	0.003	0.95	0.000
non-annotated	common to all	741.9986		NA		-0.91	0.000	-0.94	0.000	-1.19	0.000	-1.15	0.000	-1.17	0.000
non-annotated	common to all	753.3850		NA		0.71	0.009	0.75	0.005	0.67	0.012	0.94	0.001	0.87	0.001
non-annotated	common to all	758.0644		NA		-0.80	0.025	-0.91	0.009	-1.16	0.001	-1.13	0.001	-1.25	0.000
non-annotated	other (15m,1h,2h,4h)	241.9308		NA		-0.47	0.042	ND	ND	-0.54	0.013	-0.68	0.002	-1.03	0.000
non-annotated	other (15m,1h,2h,4h)	268.9896		NA		0.52	0.011	ND	ND	0.64	0.002	0.77	0.000	0.79	0.000

feature	pattern of expression	m/z	name	HMDB m/z	kegg	15min logFC	15min adj.P.Val	30 min logFC	30 min adj.P.Val	1 h logFC	1 h adj.P.Val	2 h logFC	2 h adj.P.Val	4 h logFC	4 h adj.P.Val
non-annotated	other (15m,1h,2h,4h)	336.9769		NA		1.02	0.003	ND	ND	0.73	0.030	0.70	0.035	0.97	0.003
non-annotated	other (15m,1h,2h,4h)	483.9738		NA		1.19	0.000	ND	ND	0.87	0.004	0.87	0.004	1.16	0.000
non-annotated	other (15m,2h,4h)	723.3745		NA		0.54	0.042	ND	ND	ND	ND	0.73	0.004	0.58	0.014
non-annotated	other (15m,2h,4h)	616.3907		NA		0.81	0.036	ND	ND	ND	ND	1.03	0.005	0.87	0.012
non-annotated	other (15m,2h,4h)	826.3444		NA		0.65	0.039	ND	ND	ND	ND	0.88	0.003	0.91	0.002
non-annotated	other (15m,2h,4h)	698.3615		NA		0.67	0.031	ND	ND	ND	ND	0.82	0.006	0.80	0.005
non-annotated	other (15m,2h,4h)	697.3585		NA		0.65	0.022	ND	ND	ND	ND	0.76	0.005	0.79	0.003
non-annotated	other (15m,30m,1h,2h)	285.9684		NA		-1.13	0.011	-1.03	0.020	-1.02	0.020	-0.89	0.041	ND	ND
non-annotated	other (15m,30m,1h,2h)	369.0231		NA		-3.41	0.000	-3.06	0.000	-2.25	0.002	-1.92	0.008	ND	ND
non-annotated	other (15m,30m,1h,2h)	433.9759		NA		1.30	0.000	1.37	0.000	1.24	0.000	0.81	0.004	ND	ND
non-annotated	other (15m,30m,1h,2h)	516.0196		NA		-1.15	0.010	-1.03	0.019	-1.02	0.019	-0.95	0.028	ND	ND
non-annotated	other (15m,30m,1h,2h)	580.9726		NA		1.33	0.000	1.24	0.000	1.24	0.000	0.86	0.002	ND	ND
non-annotated	other (15m,30m,1h,4h)	185.9642		NA		-0.92	0.004	-0.64	0.046	-0.77	0.013	ND	ND	-1.03	0.001
non-annotated	other (15m,30m,1h)	286.0613		NA		-1.97	0.008	-1.83	0.013	-1.63	0.026	ND	ND	ND	ND
non-annotated	other (15m,30m,1h)	286.9698		NA		0.69	0.048	0.87	0.009	0.84	0.011	ND	ND	ND	ND
non-annotated	other (15m,30m,1h)	286.9791		NA		0.91	0.004	1.18	0.000	0.97	0.002	ND	ND	ND	ND
non-annotated	other (15m,30m,1h)	287.0655		NA		-0.99	0.007	-0.89	0.015	-0.89	0.014	ND	ND	ND	ND
non-annotated	other (15m,30m,1h)	287.9824		NA		0.93	0.005	1.20	0.000	1.00	0.002	ND	ND	ND	ND
non-annotated	other (15m,30m,1h)	290.9762		NA		0.64	0.042	0.98	0.001	0.79	0.008	ND	ND	ND	ND
non-annotated	other (15m,30m,1h)	449.0321		NA		-1.14	0.027	-1.04	0.040	-1.02	0.045	ND	ND	ND	ND
non-annotated	other (15m,30m,1h)	499.9475		NA		0.95	0.000	0.72	0.006	0.79	0.002	ND	ND	ND	ND
non-annotated	other (15m,30m,2h)	352.1447		NA		1.95	0.016	1.86	0.019	ND	ND	1.80	0.023	ND	ND
non-annotated	other (15m,30m,4h)	427.0571		NA		-1.12	0.011	-0.92	0.037	ND	ND	ND	ND	1.25	0.003
non-annotated	other (15m,30m)	125.0117		NA		0.41	0.041	0.49	0.010	ND	ND	ND	ND	ND	ND
non-annotated	other (15m,30m)	126.0041		NA		0.42	0.038	0.51	0.010	ND	ND	ND	ND	ND	ND
non-annotated	other (15m,30m)	126.0126		NA		0.45	0.026	0.52	0.008	ND	ND	ND	ND	ND	ND
non-annotated	other (15m,30m)	187.0430		NA		1.50	0.010	2.13	0.000	ND	ND	ND	ND	ND	ND
non-annotated	other (15m,30m)	242.0350		NA		0.94	0.045	1.13	0.012	ND	ND	ND	ND	ND	ND
non-annotated	other (15m,30m)	249.0232		NA		0.70	0.041	0.91	0.006	ND	ND	ND	ND	ND	ND
non-annotated	other (15m,30m)	297.0207		NA		0.72	0.009	0.65	0.015	ND	ND	ND	ND	ND	ND
non-annotated	other (15m,30m)	312.9947		NA		0.79	0.050	1.12	0.004	ND	ND	ND	ND	ND	ND
non-annotated	other (15m,30m)	433.0581		NA		-1.77	0.031	-1.86	0.019	ND	ND	ND	ND	ND	ND
non-annotated	other (1h,2h,4h)	611.0924		NA		ND	ND	ND	ND	-1.19	0.038	-1.16	0.040	-1.71	0.002
non-annotated	other (1h,2h,4h)	434.0093		NA		ND	ND	ND	ND	-1.37	0.016	-1.29	0.023	-1.37	0.010
non-annotated	other (1h,2h,4h)	509.0277		NA		ND	ND	ND	ND	-1.74	0.000	-2.15	0.000	-2.31	0.000
non-annotated	other (1h,2h,4h)	542.0603		NA		ND	ND	ND	ND	-1.12	0.002	-0.74	0.047	-1.39	0.000
non-annotated	other (1h,2h,4h)	556.0501		NA		ND	ND	ND	ND	-0.85	0.013	-0.86	0.011	-1.06	0.001
non-annotated	other (1h,2h,4h)	559.0693		NA		ND	ND	ND	ND	-1.11	0.015	-1.01	0.028	-1.63	0.000
non-annotated	other (1h,2h,4h)	566.0518		NA		ND	ND	ND	ND	-1.90	0.001	-2.09	0.000	-2.50	0.000
non-annotated	other (1h,2h,4h)	581.3110		NA		ND	ND	ND	ND	0.67	0.023	0.81	0.006	0.87	0.002
non-annotated	other (1h,2h,4h)	582.3142		NA		ND	ND	ND	ND	0.64	0.048	0.76	0.016	0.82	0.006
non-annotated	other (1h,2h,4h)	684.1380		NA		ND	ND	ND	ND	0.63	0.049	1.29	0.000	1.82	0.000
non-annotated	other (1h,2h,4h)	704.1120		NA		ND	ND	ND	ND	1.77	0.000	2.36	0.000	3.03	0.000
non-annotated	other (1h,2h,4h)	794.3910		NA		ND	ND	ND	ND	0.67	0.043	0.81	0.012	1.18	0.000
non-annotated	other (1h,2h,4h)	851.1080		NA		ND	ND	ND	ND	1.89	0.000	2.57	0.000	3.36	0.000
non-annotated	other (1h,2h,4h)	867.0819		NA		ND	ND	ND	ND	0.88	0.001	1.19	0.000	1.27	0.000
non-annotated	other (1h,2h)	439.1051		NA		ND	ND	ND	ND	-0.60	0.020	-0.54	0.035	ND	ND
non-annotated	other (1h,4h)	540.0552		NA		ND	ND	ND	ND	-1.47	0.002	ND	ND	-1.91	0.000

feature	pattern of expression	m/z	name	HMDB m/z	kegg	15min logFC	15min adj.P.Val	30 min logFC	30 min adj.P.Val	1 h logFC	1 h adj.P.Val	2 h logFC	2 h adj.P.Val	4 h logFC	4 h adj.P.Val
non-annotated	other (1h,4h)	548.0410		NA		ND	ND	ND	ND	-1.04	0.032	ND	ND	-1.27	0.005
non-annotated	other (1h,4h)	468.0340		NA		ND	ND	ND	ND	-0.78	0.047	ND	ND	-0.89	0.014
non-annotated	other (1h,4h)	541.0587		NA		ND	ND	ND	ND	-1.45	0.003	ND	ND	-1.93	0.000
non-annotated	other (1h,4h)	557.0817		NA		ND	ND	ND	ND	-1.31	0.027	ND	ND	-1.76	0.002
non-annotated	other (1h,4h)	679.1019		NA		ND	ND	ND	ND	-1.27	0.042	ND	ND	-1.79	0.002
non-annotated	other (1h,4h)	696.1086		NA		ND	ND	ND	ND	-1.65	0.008	ND	ND	-1.92	0.002
non-annotated	other (1h,4h)	697.1124		NA		ND	ND	ND	ND	-1.32	0.006	ND	ND	-1.71	0.000
non-annotated	other (2h,4h)	122.9863		NA		ND	ND	ND	ND	ND	ND	0.57	0.046	0.69	0.011
non-annotated	other (2h,4h)	179.0126		NA		ND	ND	ND	ND	ND	ND	0.57	0.041	0.74	0.005
non-annotated	other (2h,4h)	197.1193		NA		ND	ND	ND	ND	ND	ND	0.62	0.041	0.75	0.009
non-annotated	other (2h,4h)	244.0273		NA		ND	ND	ND	ND	ND	ND	0.57	0.033	0.78	0.002
non-annotated	other (2h,4h)	260.0974		NA		ND	ND	ND	ND	ND	ND	0.80	0.005	0.92	0.001
non-annotated	other (2h,4h)	362.0060		NA		ND	ND	ND	ND	ND	ND	-0.98	0.010	-1.00	0.006
non-annotated	other (2h,4h)	420.2615		NA		ND	ND	ND	ND	ND	ND	0.46	0.039	0.59	0.005
non-annotated	other (2h,4h)	436.0840		NA		ND	ND	ND	ND	ND	ND	0.91	0.001	1.12	0.000
non-annotated	other (2h,4h)	572.3640		NA		ND	ND	ND	ND	ND	ND	0.72	0.013	0.73	0.008
non-annotated	other (2h,4h)	600.3953		NA		ND	ND	ND	ND	ND	ND	0.81	0.018	0.92	0.005
non-annotated	other (2h,4h)	603.3317		NA		ND	ND	ND	ND	ND	ND	0.54	0.015	0.56	0.008
non-annotated	other (2h,4h)	607.0792		NA		ND	ND	ND	ND	ND	ND	-1.22	0.004	-2.76	0.000
non-annotated	other (2h,4h)	628.0576		NA		ND	ND	ND	ND	ND	ND	-0.91	0.023	-1.80	0.000
non-annotated	other (2h,4h)	628.1272		NA		ND	ND	ND	ND	ND	ND	0.99	0.007	0.73	0.033
non-annotated	other (2h,4h)	665.1051		NA		ND	ND	ND	ND	ND	ND	0.66	0.017	0.99	0.000
non-annotated	other (2h,4h)	665.1226		NA		ND	ND	ND	ND	ND	ND	1.63	0.001	1.95	0.000
non-annotated	other (2h,4h)	666.1237		NA		ND	ND	ND	ND	ND	ND	0.59	0.049	0.90	0.002
non-annotated	other (2h,4h)	686.1013		NA		ND	ND	ND	ND	ND	ND	1.45	0.002	2.02	0.000
non-annotated	other (2h,4h)	690.1149		NA		ND	ND	ND	ND	ND	ND	0.96	0.017	1.57	0.000
non-annotated	other (2h,4h)	690.3072		NA		ND	ND	ND	ND	ND	ND	0.67	0.023	0.64	0.020
non-annotated	other (2h,4h)	693.3174		NA		ND	ND	ND	ND	ND	ND	0.49	0.037	0.60	0.007
non-annotated	other (2h,4h)	696.3567		NA		ND	ND	ND	ND	ND	ND	0.92	0.020	0.87	0.017
non-annotated	other (2h,4h)	708.1253		NA		ND	ND	ND	ND	ND	ND	0.71	0.047	1.26	0.000
non-annotated	other (2h,4h)	720.3546		NA		ND	ND	ND	ND	ND	ND	0.59	0.029	0.76	0.003
non-annotated	other (2h,4h)	724.3879		NA		ND	ND	ND	ND	ND	ND	1.02	0.022	1.11	0.008
non-annotated	other (2h,4h)	735.3645		NA		ND	ND	ND	ND	ND	ND	0.80	0.034	0.90	0.010
non-annotated	other (2h,4h)	737.3899		NA		ND	ND	ND	ND	ND	ND	0.98	0.023	1.10	0.007
non-annotated	other (2h,4h)	746.3696		NA		ND	ND	ND	ND	ND	ND	0.48	0.047	0.71	0.002
non-annotated	other (2h,4h)	749.3900		NA		ND	ND	ND	ND	ND	ND	0.52	0.031	0.53	0.017
non-annotated	other (2h,4h)	753.4218		NA		ND	ND	ND	ND	ND	ND	0.82	0.038	1.16	0.002
non-annotated	other (2h,4h)	827.3477		NA		ND	ND	ND	ND	ND	ND	0.58	0.028	0.54	0.026
non-annotated	other (2h,4h)	833.0971		NA		ND	ND	ND	ND	ND	ND	1.37	0.000	2.01	0.000
non-annotated	other (2h,4h)	863.5688		NA		ND	ND	ND	ND	ND	ND	0.80	0.040	1.09	0.004
non-annotated	other (2h,4h)	882.4069		NA		ND	ND	ND	ND	ND	ND	0.46	0.043	0.76	0.001
non-annotated	other (30m,1h,2h,4h)	524.0603		NA		ND	ND	-0.76	0.049	-1.16	0.002	-0.82	0.029	-1.31	0.000
non-annotated	other (30m,1h,2h,4h)	243.9673		NA		ND	ND	0.69	0.007	0.66	0.009	0.57	0.024	0.66	0.006
non-annotated	other (30m,1h,2h,4h)	429.0118		NA		ND	ND	-0.80	0.018	-1.05	0.002	-0.72	0.032	-1.19	0.000
non-annotated	other (30m,1h,2h,4h)	493.0538		NA		ND	ND	-1.24	0.003	-2.10	0.000	-2.46	0.000	-3.53	0.000
non-annotated	other (30m,1h,2h,4h)	494.0570		NA		ND	ND	-1.10	0.001	-1.68	0.000	-1.94	0.000	-2.09	0.000
non-annotated	other (30m,1h,2h,4h)	600.3247		NA		ND	ND	1.10	0.010	1.15	0.006	1.12	0.008	1.63	0.000
non-annotated	other (30m,1h,2h,4h)	734.1395		NA		ND	ND	-1.09	0.027	-1.56	0.001	-1.04	0.033	-2.08	0.000

feature	pattern of expression	m/z	name	HMDB m/z	kegg	15min logFC	15min adj.P.Val	30 min logFC	30 min adj.P.Val	1 h logFC	1 h adj.P.Val	2 h logFC	2 h adj.P.Val	4 h logFC	4 h adj.P.Val
non-annotated	other (30m,1h,2h)	316.0773		NA		ND	ND	0.51	0.025	0.53	0.020	0.49	0.029	ND	ND
non-annotated	other (30m,1h,4h)	576.0319		NA		ND	ND	-1.00	0.037	-1.42	0.002	ND	ND	-1.84	0.000
non-annotated	other (30m,1h,4h)	562.0372		NA		ND	ND	-0.89	0.038	-1.11	0.008	ND	ND	-1.38	0.001
non-annotated	other (30m,1h,4h)	901.5465		NA		ND	ND	0.63	0.046	0.64	0.040	ND	ND	0.62	0.029
non-annotated	other (30m,1h)	259.9414		NA		ND	ND	1.36	0.000	0.97	0.007	ND	ND	ND	ND
non-annotated	other (30m,1h)	232.8845		NA		ND	ND	-1.22	0.017	-1.14	0.026	ND	ND	ND	ND
non-annotated	other (30m,1h)	288.9830		NA		ND	ND	1.26	0.044	1.28	0.040	ND	ND	ND	ND
non-annotated	other (30m,2h)	318.0070		NA		ND	ND	0.86	0.020	ND	ND	0.96	0.008	ND	ND
non-annotated	other (30m,2h)	991.2110		NA		ND	ND	0.76	0.046	ND	ND	1.21	0.001	ND	ND
non-annotated	other (30m,4h)	312.9583		NA		ND	ND	0.92	0.017	ND	ND	ND	ND	-0.77	0.030
non-annotated	other (30m,4h)	340.9534		NA		ND	ND	1.57	0.001	ND	ND	ND	ND	-1.19	0.006
non-annotated	other (30m,4h)	366.0277		NA		ND	ND	2.25	0.045	ND	ND	ND	ND	2.31	0.023
non-annotated	unique to 1h	283.0051		NA		ND	ND	ND	ND	0.92	0.037	ND	ND	ND	ND
non-annotated	unique to 1h	284.0457		NA		ND	ND	ND	ND	-0.59	0.045	ND	ND	ND	ND
non-annotated	unique to 2h	544.3327		NA		ND	ND	ND	ND	ND	ND	0.63	0.044	ND	ND
non-annotated	unique to 2h	669.3289		NA		ND	ND	ND	ND	ND	ND	0.75	0.015	ND	ND
non-annotated	unique to 2h	699.3663		NA		ND	ND	ND	ND	ND	ND	0.64	0.017	ND	ND
non-annotated	unique to 2h	727.3985		NA		ND	ND	ND	ND	ND	ND	0.89	0.043	ND	ND
non-annotated	unique to 30m	154.9198		NA		ND	ND	-1.42	0.019	ND	ND	ND	ND	ND	ND
non-annotated	unique to 30m	216.9105		NA		ND	ND	-1.80	0.013	ND	ND	ND	ND	ND	ND
non-annotated	unique to 30m	243.9579		NA		ND	ND	-0.96	0.028	ND	ND	ND	ND	ND	ND
non-annotated	unique to 30m	245.9537		NA		ND	ND	-1.02	0.026	ND	ND	ND	ND	ND	ND
non-annotated	unique to 30m	267.9370		NA		ND	ND	1.65	0.040	ND	ND	ND	ND	ND	ND
non-annotated	unique to 30m	301.0663		NA		ND	ND	0.60	0.048	ND	ND	ND	ND	ND	ND
non-annotated	unique to 30m	302.9996		NA		ND	ND	1.01	0.010	ND	ND	ND	ND	ND	ND
non-annotated	unique to 30m	314.9740		NA		ND	ND	1.56	0.019	ND	ND	ND	ND	ND	ND
non-annotated	unique to 30m	403.9838		NA		ND	ND	2.62	0.023	ND	ND	ND	ND	ND	ND
non-annotated	unique to 30m	421.9941		NA		ND	ND	1.14	0.023	ND	ND	ND	ND	ND	ND
non-annotated	unique to 30m	714.3563		NA		ND	ND	0.99	0.021	ND	ND	ND	ND	ND	ND
non-annotated	unique to 30m	761.4036		NA		ND	ND	0.77	0.028	ND	ND	ND	ND	ND	ND
non-annotated	unique to 30m	842.3182		NA		ND	ND	0.87	0.013	ND	ND	ND	ND	ND	ND
non-annotated	unique to 30m	713.3536		NA		ND	ND	1.00	0.023	ND	ND	ND	ND	ND	ND
non-annotated	unique to 4 h	134.9863		NA		ND	ND	ND	ND	ND	ND	ND	ND	0.64	0.005
non-annotated	unique to 4h	818.5355		NA		ND	ND	ND	ND	ND	ND	ND	ND	0.94	0.041
non-annotated	unique to 4h	1071.1773		NA		ND	ND	ND	ND	ND	ND	ND	ND	-1.03	0.016
non-annotated	unique to 4h	1185.7360		NA		ND	ND	ND	ND	ND	ND	ND	ND	1.31	0.041
non-annotated	unique to 4h	152.9969		NA		ND	ND	ND	ND	ND	ND	ND	ND	0.66	0.009
non-annotated	unique to 4h	195.9931		NA		ND	ND	ND	ND	ND	ND	ND	ND	-0.80	0.001
non-annotated	unique to 4h	196.0401		NA		ND	ND	ND	ND	ND	ND	ND	ND	0.83	0.006
non-annotated	unique to 4h	209.0232		NA		ND	ND	ND	ND	ND	ND	ND	ND	0.78	0.003
non-annotated	unique to 4h	214.9527		NA		ND	ND	ND	ND	ND	ND	ND	ND	0.63	0.007
non-annotated	unique to 4h	242.0164		NA		ND	ND	ND	ND	ND	ND	ND	ND	0.40	0.041
non-annotated	unique to 4h	254.9464		NA		ND	ND	ND	ND	ND	ND	ND	ND	0.96	0.002
non-annotated	unique to 4h	256.2374		NA		ND	ND	ND	ND	ND	ND	ND	ND	0.57	0.013
non-annotated	unique to 4h	257.9736		NA		ND	ND	ND	ND	ND	ND	ND	ND	0.80	0.003
non-annotated	unique to 4h	259.9319		NA		ND	ND	ND	ND	ND	ND	ND	ND	-0.68	0.006
non-annotated	unique to 4h	261.9298		NA		ND	ND	ND	ND	ND	ND	ND	ND	-0.67	0.006
non-annotated	unique to 4h	282.2531		NA		ND	ND	ND	ND	ND	ND	ND	ND	0.78	0.022

feature	pattern of expression	m/z	name	HMDB m/z	kegg	15min logFC	15min adj.P.Val	30 min logFC	30 min adj.P.Val	1 h logFC	1 h adj.P.Val	2 h logFC	2 h adj.P.Val	4 h logFC	4 h adj.P.Val
non-annotated	unique to 4h	284.2687		NA		ND	ND	ND	ND	ND	ND	ND	ND	0.61	0.009
non-annotated	unique to 4h	285.2722		NA		ND	ND	ND	ND	ND	ND	ND	ND	0.50	0.028
non-annotated	unique to 4h	290.0893		NA		ND	ND	ND	ND	ND	ND	ND	ND	0.59	0.028
non-annotated	unique to 4h	294.0719		NA		ND	ND	ND	ND	ND	ND	ND	ND	0.56	0.022
non-annotated	unique to 4h	310.9715		NA		ND	ND	ND	ND	ND	ND	ND	ND	1.25	0.001
non-annotated	unique to 4h	325.0157		NA		ND	ND	ND	ND	ND	ND	ND	ND	0.68	0.029
non-annotated	unique to 4h	326.0440		NA		ND	ND	ND	ND	ND	ND	ND	ND	0.72	0.024
non-annotated	unique to 4h	328.0597		NA		ND	ND	ND	ND	ND	ND	ND	ND	0.90	0.006
non-annotated	unique to 4h	328.9639		NA		ND	ND	ND	ND	ND	ND	ND	ND	1.11	0.010
non-annotated	unique to 4h	334.1342		NA		ND	ND	ND	ND	ND	ND	ND	ND	0.89	0.034
non-annotated	unique to 4h	345.0733		NA		ND	ND	ND	ND	ND	ND	ND	ND	1.25	0.020
non-annotated	unique to 4h	350.1386		NA		ND	ND	ND	ND	ND	ND	ND	ND	0.63	0.021
non-annotated	unique to 4h	355.9625		NA		ND	ND	ND	ND	ND	ND	ND	ND	-1.25	0.000
non-annotated	unique to 4h	372.1193		NA		ND	ND	ND	ND	ND	ND	ND	ND	0.81	0.006
non-annotated	unique to 4h	373.9732		NA		ND	ND	ND	ND	ND	ND	ND	ND	-0.57	0.037
non-annotated	unique to 4h	388.0098		NA		ND	ND	ND	ND	ND	ND	ND	ND	2.57	0.018
non-annotated	unique to 4h	389.0132		NA		ND	ND	ND	ND	ND	ND	ND	ND	1.43	0.027
non-annotated	unique to 4h	392.2302		NA		ND	ND	ND	ND	ND	ND	ND	ND	0.56	0.011
non-annotated	unique to 4h	398.0263		NA		ND	ND	ND	ND	ND	ND	ND	ND	0.73	0.007
non-annotated	unique to 4h	412.9367		NA		ND	ND	ND	ND	ND	ND	ND	ND	0.97	0.017
non-annotated	unique to 4h	462.3003		NA		ND	ND	ND	ND	ND	ND	ND	ND	0.68	0.026
non-annotated	unique to 4h	473.3186		NA		ND	ND	ND	ND	ND	ND	ND	ND	1.40	0.005
non-annotated	unique to 4h	474.3220		NA		ND	ND	ND	ND	ND	ND	ND	ND	1.11	0.002
non-annotated	unique to 4h	481.3142		NA		ND	ND	ND	ND	ND	ND	ND	ND	0.69	0.008
non-annotated	unique to 4h	497.3187		NA		ND	ND	ND	ND	ND	ND	ND	ND	1.08	0.005
non-annotated	unique to 4h	528.2744		NA		ND	ND	ND	ND	ND	ND	ND	ND	0.82	0.037
non-annotated	unique to 4h	534.3208		NA		ND	ND	ND	ND	ND	ND	ND	ND	0.54	0.027
non-annotated	unique to 4h	535.0065		NA		ND	ND	ND	ND	ND	ND	ND	ND	0.92	0.042
non-annotated	unique to 4h	579.2954		NA		ND	ND	ND	ND	ND	ND	ND	ND	0.49	0.033
non-annotated	unique to 4h	585.0767		NA		ND	ND	ND	ND	ND	ND	ND	ND	-1.70	0.026
non-annotated	unique to 4h	586.0803		NA		ND	ND	ND	ND	ND	ND	ND	ND	-1.20	0.021
non-annotated	unique to 4h	592.2142		NA		ND	ND	ND	ND	ND	ND	ND	ND	0.74	0.015
non-annotated	unique to 4h	601.3265		NA		ND	ND	ND	ND	ND	ND	ND	ND	1.01	0.003
non-annotated	unique to 4h	633.1280		NA		ND	ND	ND	ND	ND	ND	ND	ND	0.67	0.037
non-annotated	unique to 4h	644.1480		NA		ND	ND	ND	ND	ND	ND	ND	ND	-0.73	0.044
non-annotated	unique to 4h	665.0700		NA		ND	ND	ND	ND	ND	ND	ND	ND	-0.82	0.041
non-annotated	unique to 4h	668.1138		NA		ND	ND	ND	ND	ND	ND	ND	ND	-1.41	0.015
non-annotated	unique to 4h	671.0867		NA		ND	ND	ND	ND	ND	ND	ND	ND	0.70	0.017
non-annotated	unique to 4h	672.1043		NA		ND	ND	ND	ND	ND	ND	ND	ND	0.75	0.013
non-annotated	unique to 4h	687.0518		NA		ND	ND	ND	ND	ND	ND	ND	ND	-0.83	0.047
non-annotated	unique to 4h	700.0801		NA		ND	ND	ND	ND	ND	ND	ND	ND	-1.51	0.008
non-annotated	unique to 4h	703.0257		NA		ND	ND	ND	ND	ND	ND	ND	ND	-1.48	0.000
non-annotated	unique to 4h	705.1151		NA		ND	ND	ND	ND	ND	ND	ND	ND	0.91	0.000
non-annotated	unique to 4h	725.0076		NA		ND	ND	ND	ND	ND	ND	ND	ND	-1.27	0.001
non-annotated	unique to 4h	745.3111		NA		ND	ND	ND	ND	ND	ND	ND	ND	0.66	0.031
non-annotated	unique to 4h	766.0672		NA		ND	ND	ND	ND	ND	ND	ND	ND	1.00	0.001
non-annotated	unique to 4h	775.0540		NA		ND	ND	ND	ND	ND	ND	ND	ND	-0.84	0.012
non-annotated	unique to 4h	788.0489		NA		ND	ND	ND	ND	ND	ND	ND	ND	1.03	0.001

feature	pattern of expression	m/z	name	HMDB m/z	kegg	15min logFC	15min adj.P.Val	30 min logFC	30 min adj.P.Val	1 h logFC	1 h adj.P.Val	2 h logFC	2 h adj.P.Val	4 h logFC	4 h adj.P.Val
non-annotated	unique to 4h	794.5356		NA		ND	ND	ND	ND	ND	ND	ND	ND	0.97	0.027
non-annotated	unique to 4h	795.3951		NA		ND	ND	ND	ND	ND	ND	ND	ND	0.75	0.005
non-annotated	unique to 4h	806.0583		NA		ND	ND	ND	ND	ND	ND	ND	ND	0.83	0.002
non-annotated	unique to 4h	853.4416		NA		ND	ND	ND	ND	ND	ND	ND	ND	1.37	0.028
non-annotated	unique to 4h	854.4451		NA		ND	ND	ND	ND	ND	ND	ND	ND	1.39	0.022
non-annotated	unique to 4h	855.4601		NA		ND	ND	ND	ND	ND	ND	ND	ND	0.88	0.044
non-annotated	unique to 4h	856.4624		NA		ND	ND	ND	ND	ND	ND	ND	ND	0.68	0.024
non-annotated	unique to 4h	864.5714		NA		ND	ND	ND	ND	ND	ND	ND	ND	0.67	0.007
non-annotated	unique to 4h	872.4555		NA		ND	ND	ND	ND	ND	ND	ND	ND	0.56	0.024
non-annotated	unique to 4h	894.5141		NA		ND	ND	ND	ND	ND	ND	ND	ND	0.79	0.018
non-annotated	unique to 4h	927.4780		NA		ND	ND	ND	ND	ND	ND	ND	ND	0.76	0.015
non-annotated	unique to 4h	1186.7393		NA		ND	ND	ND	ND	ND	ND	ND	ND	1.13	0.041
non-annotated	unique to 4h	1189.7073		NA		ND	ND	ND	ND	ND	ND	ND	ND	0.72	0.031
non-annotated	unique to 4h	1207.7181		NA		ND	ND	ND	ND	ND	ND	ND	ND	1.82	0.009
non-annotated	unique to 4h	1208.7214		NA		ND	ND	ND	ND	ND	ND	ND	ND	1.77	0.008
non-annotated	unique to 4h	1209.7293		NA		ND	ND	ND	ND	ND	ND	ND	ND	1.24	0.013

Supplementary Table S5. Partial list of annotated DE features at 15 min of ischemia (the numbers in the last column correspond to the numbers in Fig. 4a (main text of the article) and Table S6); statistical calculations were done using MetaboAnalyst software, FDR<0.05.

Name	m/z	log2FoldChange	FDR	#
(S)-NADHX	682.1294	2.63	0.0005582	31
S-Cysteinosuccinic acid	236.0243	2.31	0.0000324	9
L-Glutamic acid (Glutamate)	146.0469	0.97	0.0100670	3
5-Phosphoribosyl-4-carboxy-5-aminoimidazole	677.0918	0.74	0.0265290	30
Glycerol 3-phosphate	171.0074	0.73	0.0127000	6
D-glucuronic acid 1-phosphate	273.0010	0.69	0.0004689	13
Cysteic acid	167.9983	0.51	0.0129460	5
Taurine	124.0083	0.40	0.0083334	1
Inosinic acid (IMP)	347.0410	-4.27	0.0000023	49
Citric acid	191.0209	-3.37	0.0000002	37
Guanosine diphosphate/8-oxo-dGDP	442.0184	-3.27	0.0000054	59
Adenosine triphosphate (ATP)	505.9898	-3.23	0.0001276	62
Fructose 1,6-bisphosphate/1D-Myo-inositol 1,3-bisphosphate/ Alpha-D-Glucose 1,6-bisphosphate	338.9900	-2.84	0.0006155	45
Uridine diphosphate glucose	565.0509	-2.57	0.0006321	65
ADP	426.0235	-2.42	0.0000735	55
Cytidine diphosphate (CDP)	402.0122	-2.21	0.0007436	51
Gossypetin 8-glucuronide 3-sulfate	573.0202	-2.20	0.0001254	66
Guanosine triphosphate (GTP)	521.9847	-2.03	0.0001202	63
Uridine 5'-diphosphate (UDP)	402.9963	-1.93	0.0011598	52
IDP	427.0094	-1.92	0.0000186	56
Cyclic GMP	344.0413	-1.92	0.0000025	46
L-aspartic acid	132.0312	-1.85	0.0007811	33
Adenine	134.0482	-1.62	0.0000156	34
1-Methoxyspirobrassinin	279.0279	-1.56	0.0005265	40
Cyclic adenosine monophosphate (cAMP)	328.0463	-1.54	0.0001706	43
6-(2-carboxy-5-hydroxyphenoxy)-3,4,5-trihydroxyoxane-2-carboxylic acid	329.0508	-1.49	0.0000779	44
ADP-ribose 1"-2" cyclic phosphate	620.0215	-1.40	0.0025957	67
Guanosine monophosphate (GMP)	362.0520	-1.39	0.0000077	50
Diphosphate (Pyrophosphate)	176.9370	-1.35	0.0002622	36
Uridine triphosphate (UTP)	482.9626	-1.07	0.0208280	61
Adenosine monophosphate (AMP)	346.0585	-0.80	0.0014674	48
ADP-ribose 2'-phosphate	638.0321	-0.76	0.0046955	68
Cytidine monophosphate (CMP)	322.0505	-0.69	0.0021554	42

Supplementary Table S6. List of annotated features that were differentially expressed during the initial 1 h of ischemia (i.e. at 15 min, 30 min, or 1 h).

#	m/z	HMBD name	HMDB adduct m/z	regulation	DE time points
1	124.0083	Taurine	124.0074	up	15min, 30min
2	125.0077	2-Acetylthiophene	125.0067	up	15min, 30min
3	146.0469	L-Glutamic acid (Glutamate)	146.0459	up	15min, 30min, 1h
4	151.0273	Xanthine	151.0261	up	1h, 4h
5	167.9983	Cysteic acid	167.9972	up	15min, 30min
6	171.0074	Glycerol 3-phosphate	171.0064	up	15min, 30min, 2h, 4h
7	207.0452	Cysteinyl-Serine	207.0445	up	30min
8	230.0116	Benzeneacetamide-4-O-sulphate	230.0129	up	30min, 1h, 2h, 4h
9	236.0243	S-Cysteinosuccinic acid	236.0234	up	15min, 30min, 1h, 2h, 4h
10	271.0148	1,3,5-trihydroxy-4-(sulfooxy)cyclohexane-1-carboxylic acid	271.0129	up	15min, 30min, 1h, 2h, 4h
11	271.0245	[2-methoxy-4-(3-oxobut-1-en-1-yl)phenyl]oxidanesulfonic acid	271.0282	up	15min, 30min, 1h, 2h, 4h
12	272.0084	Stepronin	272.0057	up	15min, 30min, 1h, 2h, 4h
13	273.0010	D-glucuronic acid 1-phosphate	273.0017	up	15min, 30min, 1h, 2h, 4h
14	293.0436	Wasalexin A	293.0424	up	15min, 30min, 1h, 2h, 4h
15	297.0392	Irilone	297.0405	up	1h, 2h, 4h
16	300.0898	Citrusinine I	300.0877	up	15min, 30min, 1h, 2h, 4h
17	315.0498	Isorhamnetin	315.0510	up	15min, 30min, 1h, 2h, 4h
18	316.0638	5,7,18,20-Tetraoxa-24-azahexacyclo[11.11.0.0Â²,Â¹â°.0â´,â¸.0Â¹â´,Â²Â².0Â¹â·,Â²Â¹]tetracosa-1(24),2,4(8),9,11,13,15,17(21),22-nonaene	316.0615	up	30min
19	324.9794	Niclosamide	324.9788	up	30min
20	335.0663	S-Nitrosoglutathione	335.0667	up	1h, 2h, 4h
21	352.9509	Pipobroman	352.9506	up	15min, 30min, 1h
22	365.0922	Atovaquone	365.0950	up	30min
23	415.0326	5-{[(2E)-3-(3,4-dihydroxyphenyl)prop-2-enoyl]oxy}-4-hydroxy-3-(sulfooxy)cyclohex-1-ene-1-carboxylic acid	415.0341	up	1h, 2h, 4h
24	445.0543	CDP-Ethanolamine	445.0531	up	30min, 2h
25	463.2843	1-(11Z-eicosenoyl)-glycero-3-phosphate	463.2830	up	30min, 4h
26	571.2903	1-Palmitoylglycerophosphoinositol	571.2889	up	30min, 1h, 4h
27	597.3061	1-Oleoylglycerophosphoinositol	597.3045	up	1h, 2h, 4h
28	599.3215	LysoPI(18:0/0:0)	599.3202	up	30min, 1h, 2h, 4h
29	627.1235	(3-{3,5,7-trihydroxy-8-[2-hydroxy-3-(3-hydroxyphenyl)-1-(2,4,6-trihydroxyphenyl)propyl]-3,4-dihydro-2H-1-benzopyran-2-yl}phenyl)oxidanesulfonic acid	627.1178	up	30min, 1h, 2h, 4h
30	677.0918	5-Phosphoribosyl-4-carboxy-5-aminoimidazole	677.0917	up	15min, 4h
31	682.1294	(S)-NADHX	682.1281	up	15min, 30min, 1h, 2h, 4h
32	859.5361	Pi(16:0/20:3(8Z,11Z,14Z))	859.5342	up	1h, 2h, 4h
33	132.0312	L-aspartic acid	132.0302	down	15min, 30min, 1h, 2h, 4h
34	134.0482	Adenine	134.0472	down	15min, 30min, 1h, 2h, 4h
35	138.9812	Phosphonoacetic acid	138.9802	down	1h, 2h, 4h
36	176.9370	Diphosphate (Pyrophosphate)	176.9360	down	15min, 30min, 1h, 2h, 4h
37	191.0209	Citric acid	191.0197	down	15min, 30min, 1h, 2h, 4h
38	238.8928	Trimetaphosphoric acid	238.8917	down	15min, 30min, 1h, 2h, 4h
39	272.9582	Ribose-1-arsenate	272.9597	down	15min, 30min, 1h, 2h, 4h

#	m/z	HMBD name	HMDB adduct m/z	regulation	DE time points
40	279.0279	1-Methoxyspirobrassinin	279.0267	down	15min, 30min, 1h, 2h, 4h
41	305.0245	2-(3,4-Dihydroxybenzoyloxy)-4,6-dihydroxybenzoic acid	305.0222	down	15min, 30min, 1h, 2h, 4h
42	322.0505	Cytidine monophosphate (18ppm)	322.0446	down	15min, 1h, 2h, 4h
43	328.0463	Cyclic AMP	328.0452	down	15min, 30min, 1h, 2h, 4h
44	329.0508	6-(2-carboxy-5-hydroxyphenoxy)-3,4,5-trihydroxyoxane-2-carboxylic acid	329.0489	down	15min, 30min, 1h, 2h, 4h
45	338.9900	Fructose 1,6-bisphosphate/1D-Myo-inositol 1,3-bisphosphate/ Alpha-D-Glucose 1,6-bisphosphate	338.9888	down	15min, 30min, 1h, 2h, 4h
46	344.0413	Cyclic GMP	344.0402	down	15min, 30min, 1h, 2h, 4h
47	346.0473	5'-Hydroxypiroxicam	346.0463	down	30min, 1h, 2h, 4h
48	346.0585	Adenosine monophosphate (AMP)	346.0558	down	15min, 30min, 1h, 2h, 4h
49	347.0410	Inosinic acid (IMP)	347.0398	down	15min, 30min, 1h, 2h, 4h
50	362.0520	Guanosine monophosphate (GMP)	362.0507	down	15min, 30min, 1h, 2h, 4h
51	402.0122	Cytidine diphosphate (CDP)	402.0109	down	15min, 30min, 1h, 2h, 4h
52	402.9963	Uridine 5'-diphosphate (UDP)	402.9949	down	15min, 30min, 1h, 2h, 4h
53	411.0013	[2-hydroxy-5-(3,5,7-trihydroxy-6-methoxy-4-oxo-4H-chromen-2-yl)phenyl]oxidanesulfonic acid	411.0028	down	1h, 2h, 4h
54	426.0131	Adenosine phosphosulfate	426.0126	down	15min, 30min, 1h, 2h, 4h
55	426.0235	Adp	426.0221	down	15min, 30min, 1h, 2h, 4h
56	427.0094	IDP	427.0062	down	15min, 30min, 1h, 2h, 4h
57	427.0309	{4-[2,3-dioxo-3-(2,4,6-trihydroxy-3-methoxyphenyl)propyl]-2-methoxyphenyl}oxidanesulfonic acid	427.0341	down	15min, 30min, 1h, 2h, 4h
58	428.0276	Cloransulam-methyl	428.0237	down	15min, 30min, 1h, 2h, 4h
59	442.0184	Guanosine diphosphate/8-oxo-dGDP	442.0171	down	15min, 30min, 1h, 2h, 4h
60	474.0018	Dideoxyadenosine Triphosphate	473.9986	down	1h, 2h, 4h
61	482.9626	Uridine triphosphate (UTP)	482.9613	down	15min, 30min, 1h, 2h, 4h
62	505.9898	Adenosine triphosphate (ATP)	505.9885	down	15min, 30min, 1h, 2h, 4h
63	521.9847	Guanosine triphosphate (GTP)	521.9834	down	15min, 30min, 1h, 2h, 4h
64	558.0658	Adenosine diphosphate ribose	558.0644	down	1h, 2h, 4h
65	565.0509	Uridine diphosphate glucose	565.0477	down	15min, 30min, 1h, 2h, 4h
66	573.0202	Gossypetin 8-glucuronide 3-sulfate	573.0192	down	15min, 30min, 1h, 2h, 4h
67	620.0215	ADP-ribose 1''-2'' cyclic phosphate	620.0201	down	15min, 30min, 1h, 2h, 4h
68	638.0321	ADP-ribose 2'-phosphate	638.0307	down	15min, 30min, 1h, 2h, 4h
69	678.0981	UDP-N-acetylmuraminate	678.0954	down	1h, 4h
70	784.1511	FAD	784.1499	down	30min, 1h
71	786.1671	FADH	786.1655	down	30min, 1h, 2h

================ END of SUPPLEMENTRY MATERIAL ================
